# A comprehensive long-read isoform analysis platform and sequencing resource for breast cancer

**DOI:** 10.1126/sciadv.abg6711

**Published:** 2022-01-19

**Authors:** Diogo F. T. Veiga, Alex Nesta, Yuqi Zhao, Anne Deslattes Mays, Richie Huynh, Robert Rossi, Te-Chia Wu, Karolina Palucka, Olga Anczukow, Christine R. Beck, Jacques Banchereau

**Affiliations:** 1 The Jackson Laboratory for Genomic Medicine, Farmington, CT 06032 USA.; 2 Department of Genetics and Genome Sciences, University of Connecticut Health Center, Farmington, CT 06030, USA.; 3 Institute for Systems Genomics, University of Connecticut Health Center, Farmington, CT 06030, USA.

## Abstract

Tumors display widespread transcriptome alterations, but the full repertoire of isoform-level alternative splicing in cancer is unknown. We developed a long-read (LR) RNA sequencing and analytical platform that identifies and annotates full-length isoforms and infers tumor-specific splicing events. Application of this platform to breast cancer samples identifies thousands of previously unannotated isoforms; ~30% affect protein coding exons and are predicted to alter protein localization and function. We performed extensive cross-validation with -omics datasets to support transcription and translation of novel isoforms. We identified 3059 breast tumor–specific splicing events, including 35 that are significantly associated with patient survival. Of these, 21 are absent from GENCODE and 10 are enriched in specific breast cancer subtypes. Together, our results demonstrate the complexity, cancer subtype specificity, and clinical relevance of previously unidentified isoforms and splicing events in breast cancer that are only annotatable by LR-seq and provide a rich resource of immuno-oncology therapeutic targets.

## Teaser

Breast cancer long-read transcriptome profiling reveals patient-enriched isoforms, with implications for disease progression.

## INTRODUCTION

Transcriptomic and proteomic diversity are influenced by alternative splicing (AS), transcription initiation, and polyadenylation in healthy and diseased cells (*1*). Human tumors, including breast cancers, exhibit widespread changes in the AS isoform repertoire (*2*–*4*), caused either by somatic mutation or mis-expression of the splicing regulatory machinery (*5*). Specific spliced isoforms are important for cancer initiation, progression, metastasis, and drug resistance, with some AS events significantly linked to patient survival (*5*). For example, splicing of *CD44*, a transmembrane glycoprotein that functions in cell division, viability, and adhesion, has been linked with tumor progression and epithelial-to-mesenchymal transition in breast and ovarian cancer models (*6*, *7*). Although the effects of a handful of spliced isoforms in cancer have been studied (*5*), the clinical relevance of most isoform switches in tumors remains poorly characterized.

Global analyses of cancer transcriptomes have cataloged AS profiles in oncogenesis using short-read RNA sequencing (RNA-seq) data and have identified a number of recurrent and tumor-specific splicing alterations across many cancer types, including breast (*2*, *3*, *8*–*10*). The detection and quantification of AS events using short-read RNA-seq data are inherently dependent on alignment of the RNA-seq fragments to a reference genome and applying algorithmic reconstruction to identify cancer-associated isoforms. However, this approach often yields only a partial view of the splicing repertoire because of limitations of transcript assembly tools. Current state-of-the-art spliced isoform reconstruction methods can only assemble ~20 to 40% of human transcriptomes (*11*, *12*). Therefore, approaches that exclusively use short-read RNA-seq data are unable to fully characterize the cancer-associated AS isoform landscape, including the discovery of novel spliced isoforms involving nonadjacent exons.

Long-read mRNA sequencing (LR-seq) is able to accurately capture full-length (FL) isoforms from start to end, eliminating the need for reference-based transcript reconstruction (*11*–*15*). LR-seq of human and mouse cell and tissue transcriptomes has revealed a rich diversity of spliced isoforms (*16*–*21*). In cancer research, the use of LR-seq to identify primary tumor-associated spliced isoforms remains underexploited and has been limited to the study of human leukemia samples (*22*, *23*). The ability to aquire the depth of coverage needed to accurately quantitate transcripts using LR data is prohibitively expensive. Therefore, there is a need for a systematic application of LR-seq and subsequent analysis with short read RNA-seq to provide a more comprehensive view of the complexity of transcriptomes in primary tumors.

We use LR-seq and a multilevel analytical platform to thoroughly characterize the AS isoform landscape in breast cancer and normal breast samples. Our analyses identified tumor-specific isoforms, including isoforms associated with poor survival and specific breast cancer subtypes, and provide a library of novel breast tumor–specific isoforms as a resource for immuno-oncology therapeutic development.

## RESULTS

### LR-seq uncovers thousands of previously unidentified isoforms in human breast tumors

**Fig. 1.**
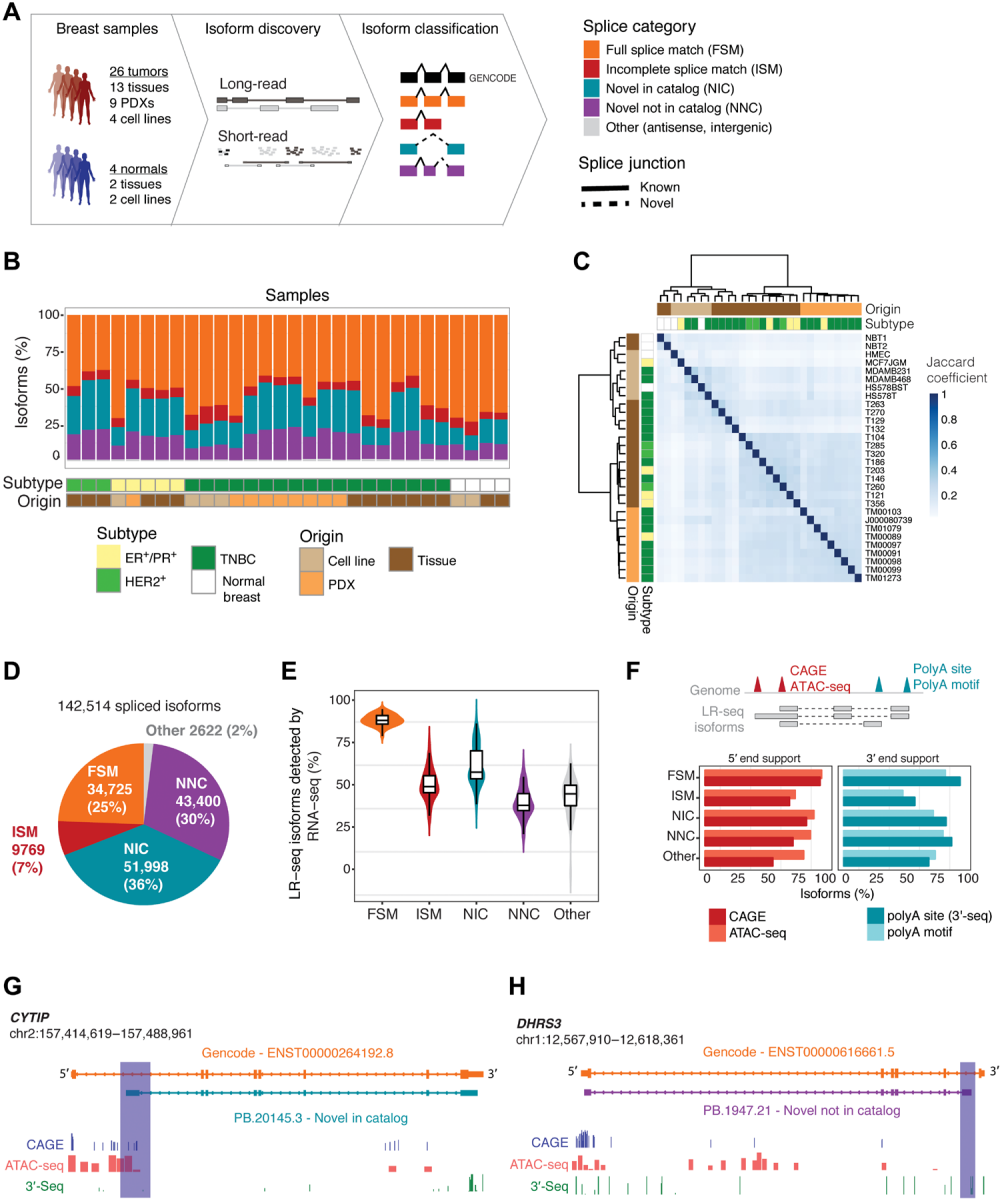
LR-seq identifies previously undetected isoforms in breast cancer. (**A**) Schematic of breast cancer isoform profiling by LR-seq and short-read RNA-seq. LR-seq isoforms are classified on the basis of their similarity to GENCODE isoforms using SQANTI isoform structural categories (see legend). Novel splice junctions are depicted by dashed lines and known junctions by solid lines. See also fig. S1 and file S1. (**B**) LR-seq isoforms detected in individual breast cancer or normal samples are colored by categories from (A), show per tissue subtype and origin. See also file S2. (**C**) Hierarchical clustering of samples profiled by LR-seq based on the Jaccard pairwise similarity coefficient. (**D**) Classification of LR-seq isoforms from merged tumor and normal samples from (B). The percent and number of distinct isoforms in each category from (A) are indicated. See also figs. S2 and S3. (**E**) Percent of LR-seq isoforms detected by RNA-seq in 29 breast cancer and normal samples, plotted per category from (A). (**F**) Percent of LR-seq isoform transcription start sites supported by CAGE (FANTOM5) or ATAC-seq (TCGA breast) peaks, transcription termination sites supported by the presence of a poly(A) motif (SQANTI2), or 3′-seq peaks from the polyA site database, plotted per category from (A). The diagram at the top exemplifies isoforms with first exons (5′ ends) validated by CAGE or ATAC-seq peaks, and terminal exons (3′ end) supported by 3′-seq peaks or poly(A) motifs. (**G** and **H**) Structure of *CYTIP* (G) or *DHRS3* (H) previously unidentified LR-seq isoforms compared to GENCODE isoforms, along with CAGE or ATAC-seq support for unknown transcription start site (G) and 3′-seq peaks supporting the previously unknown transcription termination site (H). Novel regions are highlighted.

To interrogate the AS isoform landscape of breast cancer, we performed LR-seq on four normal human breast and 26 tumor samples. Our normal samples consisted of two cell lines and two primary tissues, and our breast cancer samples included 13 primary human breast tumor biopsies [three hormone-positive, *ER*^+^/*PR*^+^; three *HER2*^+^; and seven triple-negative, TNBC (triple negative breast cancer)], nine patient-derived xenograft (PDX) tumors, and four cancer cell lines (Fig. 1A and file S1).

Isoforms obtained with single-molecule real time (SMRT) circular consensus sequencing (CCS) using the PacBio RSII and Sequel platforms were polished using the ToFU (Transcript Isoforms Full length and Unassembled) pipeline (Materials and Methods and fig. S1). An FL isoform consists of a single-mRNA molecule containing a polyadenylation [poly(A)] tail, where the entire transcript including cDNA adaptors at the 5′ and 3′ ends are successfully sequenced. After ToFU consensus clustering, 84% of CCS reads achieve 99.999% (Q50) accuracy (file S2). Overall, per library, we obtained an average of 546,000 CCS reads, which after processing resulted in ~21,000 FL polished isoforms (file S2). As a quality control step after ToFU, we filtered transcripts with inadequate splice junction support and those that contained signatures of poly(A) intrapriming or noncanonical junctions derived from reverse transcriptase template switching (Materials and Methods and fig. S1).

Next, isoforms were classified into known or novel isoforms based on their splice junction match to a reference transcriptome (GENCODE v.30) using SQANTI. Known isoforms are classified as full-splice match (FSM), while novel isoforms include both transcripts that harbor a combination of known splice donors or acceptors that have not been previously cataloged in the same transcript [novel in catalog (NIC)] and isoforms containing at least one splice site not present in GENCODE v.30 [novel not in catalog (NNC)] (Fig. 1A). Overall, novel isoforms account for 17 to 55% of sequenced transcripts in the individual samples (average = 37%; Fig. 1B). We performed sample clustering based on the Jaccard pairwise similarity coefficient, which measured the degree of overlap in isoforms detected among samples profiled by LR-seq (Fig. 1C). Overall, tumors clustered separately from normal breast, with the exception of HS578BST, a nontransformed cell line that clustered with its paired tumor-derived cell line from the same patient (HS578T). In addition, tumor samples clustered mostly by origin rather than breast cancer subtype, with tumors derived from PDX and primary tissues sharing a higher degree of similarity when compared to those of cell line origin (Fig. 1C). Thus, tumors derived from clinical samples expressed isoforms that cannot be captured in cell lines. The proportion of NIC and NNC isoforms is ~2-fold higher in all tumor subtypes versus normal samples (fig. S2A). Last, we constructed an LR-seq breast cancer transcriptome by merging the 30 individual samples and removing redundant isoforms.

Our comprehensive LR-seq breast cancer transcriptome contains 142,514 unique FL transcript isoforms (Fig. 1D) spanning 16,772 annotated genes and 905 unknown loci, with a mean isoform length of 2.6 kb (fig. S2B). Only a small fraction (2%) of poly(A)–sequenced transcripts were previously undetected antisense transcripts or mapped to intergenic regions (Fig. 1C). Two-thirds of the breast cancer LR-seq isoforms were novel (NIC or NNC) (Fig. 1D), and the majority of novel tumor isoforms (81%) originated from patient samples, thus denoting their relevance for studying primary breast cancer (fig. S2C). Also, novel isoforms occurred at a higher frequency in tumors derived from primary tissues (NIC = 37%, NNC = 31%) when compared to tumors originated from cell lines and PDXs (NIC = 26%, NNC = 16%; table S1). Within these NIC and NNC isoforms, LR-seq identified 67,727 unique splice junctions across 14,490 genes that were not previously annotated in GENCODE (fig. S2D). The guanine-cytosine (GC) content adjacent to previously unidentified splice sites was higher than the known junction regions, suggesting that junctions in GC-rich regions may be underrepresented when using traditional sequencing platforms (fig. S2E). There was a positive correlation between the number of exons and number of novel LR-seq isoforms (fig. S3), denoting that genes with higher exon complexity tend to generate a higher isoform repertoire. Last, a large fraction of NIC (58%) and NNC (73%) isoforms were detected in only a single sample (fig. S2F), while 19% of FSM isoforms are sample specific. This may indicate that novel isoforms arise because of tumor heterogeneity and lack of coverage saturation in individual samples. Overall, breast cancer LR-seq identifies thousands of spliced isoforms that are not represented in current transcript databases.

### Breast cancer LR-seq isoforms are supported by orthogonal -omics data

To assess the support for LR-seq isoforms by short-read sequencing, we performed RNA-seq and quantified isoform expression in 29 of our 30 LR-seq profiled breast samples. Briefly, 76–base pair long paired-end RNA-seq libraries were sequenced at an average depth of 46 million reads per sample and mapped to our LR-seq breast cancer transcriptome using hisat2 and quantified using StringTie. While 89% of the annotated isoforms (FSM) were detected by RNA-seq [FPKM (fragments per kilobase per million mapped reads) > 0.5], NIC and NNC isoforms have average detection rates of 62 and 41%, respectively (Fig. 1E).

In addition to RNA-seq, we used multiple orthogonal datasets to assess the reliability of previously unidentified breast cancer LR-seq isoforms, including CAGE (cap analysis gene expression), ATAC-seq (assay for transposase-accessible chromatin using sequencing), and 3′-seq. Previously unidentified 5′ isoform regions substantially overlapped with CAGE-validated transcription start sites (FANTOM5 CAGE) and open chromatin regions detected by ATAC-seq in The Cancer Genome Atlas (TCGA) breast cancer tumors (Fig. 1F) (*24*). Similarly, 3′ ends of novel LR-seq isoforms were supported by poly(A) motifs detected by SQANTI2 and bona fide transcription termination sites mapped using 3′-seq assays obtained from the poly(A) site database (Fig. 1F). For example, our LR-seq breast cancer transcriptome identified a novel *CYTIP* isoform originating from an alternative transcription start site supported by proximal CAGE and ATAC-seq peaks (Fig. 1G). We also found a *DHRS3* isoform with a novel termination site supported by 3′-seq (Fig. 1H).

Altogether, the integration of LR-seq with orthogonal data reveals that ~80% of our previously unidentified (NIC and NNC) breast cancer isoforms are validated by genomics (ATAC-seq) and/or transcriptomics (CAGE, 3′-seq) across independent samples.

### Breast cancer oncogenes and pathways are enriched in previously unidentified spliced isoforms

**Fig. 2.**
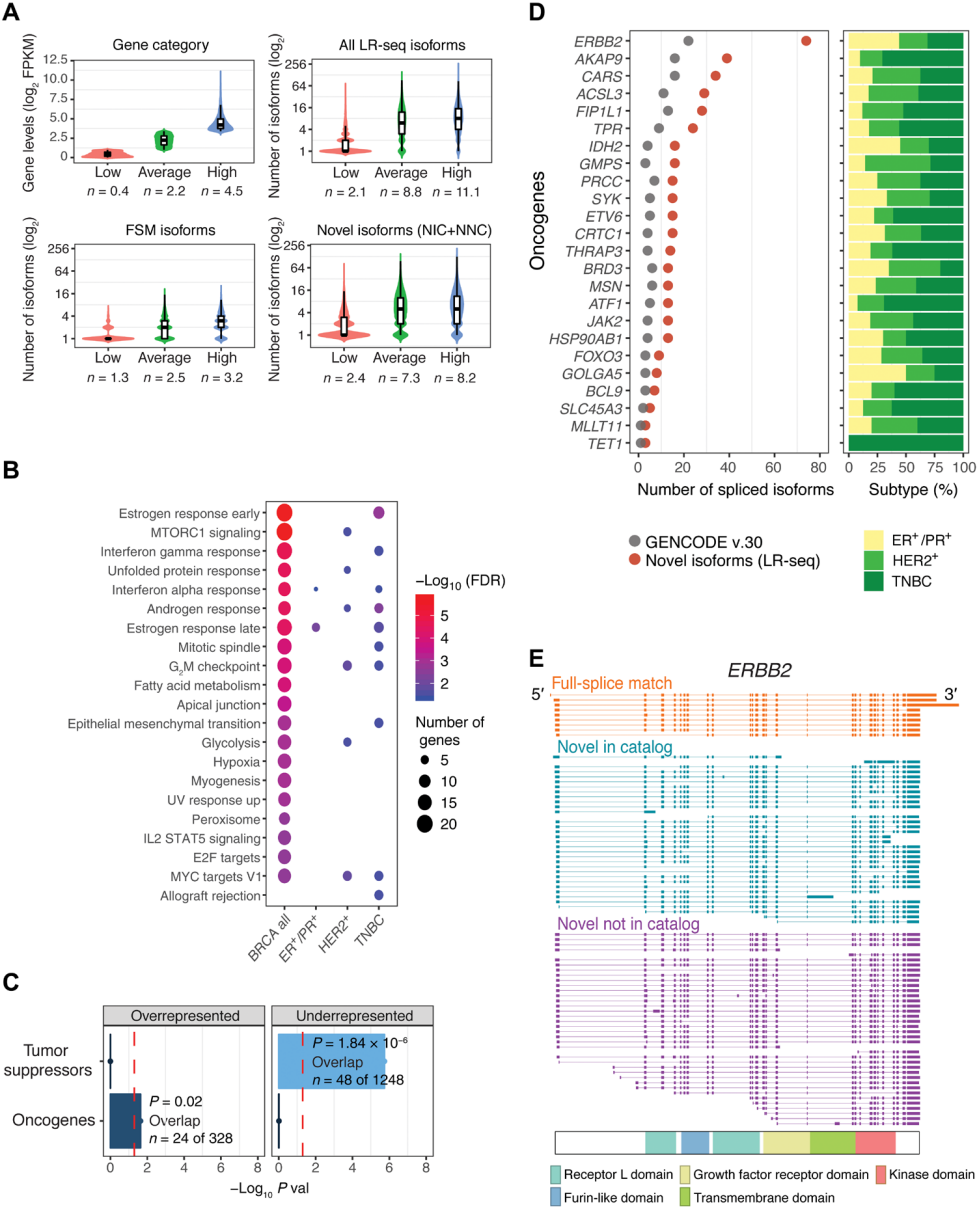
Previously unidentified LR-seq isoforms detected in breast tumors are enriched in cancer-associated pathways and oncogenes. (**A**) Correlation between gene expression levels from RNA-seq and number of transcript isoforms detected by LR-seq. Genes are binned on the basis of quartile expression: low (first quartile), average (second and third quartiles), and high (fourth quartile); where *n* is the mean log_2_ FPKM expression. Distribution of isoform numbers for each gene bin; where *n* is the mean absolute number of isoforms in the category. (**B**) Pathways significantly enriched [MSigDB, false discovery rate (FDR) < 0.05)] for genes with novel isoforms detected by LR-seq in all breast tumors or specific subtypes (HER2^+^, ER^+^/PR^+^, and TNBC). Bubble size denotes the number of genes with novel isoforms in each pathway, and color denotes significance. See also fig. S4A. (**C**) Enrichment analysis of oncogenes and tumor suppressors in genes with unannotated isoforms detected by LR-seq (hypergeometric test, *P* < 0.05, cutoff indicated by a red dotted line). Oncogenes and tumor suppressor gene lists are obtained from MSigDB and TSGene databases, respectively. (**D**) Number of novel LR-seq isoforms compared to annotated GENCODE isoforms for selected oncogenes (left). Barplots (right) indicate the tumor subtypes (colored as in Fig. 1B) where novel isoforms were detected. (**E**) Structure of LR-seq *ERBB2* isoforms detected in breast tumors, grouped by isoform structural category from Fig. 1A. Included exons or introns are represented by solid boxes, spliced introns or exons by a line. The localization of ERBB2 protein domains is indicated.

To assess the importance of novel isoforms from our LR-seq breast cancer transcriptome, we first examined the expression levels and gene pathways associated with these transcripts. Genes were binned into three groups based on our RNA-seq expression levels: low, average, and high based on FPKM cutoffs (Fig. 2A). Novel isoforms (NIC + NNC) were detected at similar rates for genes expressed at average and high levels (Fig. 2A) and at a lower rate for the lowest expressed genes, similar to FSM isoforms from GENCODE v.30. These data indicate that LR-seq detected transcripts even for lowly expressed genes and that NIC and NNC isoforms from our LR-seq data are expressed at appreciable levels.

Next, we rank-ordered genes based on their ratio of isoform number gain when compared with GENCODE v.30 (#NIC + NNC isoforms/#GENCODE) and selected genes with >2-fold increase for pathway enrichment analysis. We performed this analysis for all combined breast tumor isoforms and for isoforms in individual breast cancer subtypes to identify pathways that are common or specific to breast cancer subtypes (fig. S2A). When combining all tumors by subtype, spliced genes with novel isoforms are strongly associated with key breast cancer pathways, including estrogen, androgen, and interferon gamma response, mTORC1 (mammalian target of rapamycin complex 1) signaling, and mitotic spindle regulation (Fig. 2B and fig. S4A). Other cancer relevant pathways are also overrepresented such as metabolism (glycolysis, hypoxia, and fatty acid metabolism), replication (mitotic spindle and G_2_M checkpoint pathways), and development (myogenesis and EMT). Some cancer-related pathways were found to be enriched in a specific subtype such as glycolysis and mTORC1 signaling in HER2^+^ tumors, while others were shared. Myc targets were enriched in both HER2^+^ and TNBC tumors, while estrogen response was common to ER^+^/PR^+^ and TNBC. Notably, oncogenes are significantly overrepresented when isoforms from all tumors are combined (Fig. 2C), while tumor suppressors are underrepresented in this gene set (Fig. 2C).

We next examined individual genes that had a high gain of novel splice isoforms in our LR-seq breast cancer transcriptome. In total, 24 oncogenes including the human epidermal growth factor receptor 2 (*ERBB2*) exhibit a twofold increase in NIC + NNC isoforms compared to GENCODE v.30 (Fig. 2D). *ERBB2* is often overexpressed in breast cancer due to gene amplification, and at least three spliced isoforms with clinical relevance have been identified (*25*, *26*). In addition to the nine isoforms in GENCODE v.30, we detected 36 NIC and 38 NNC distinct spliced isoforms, revealing the complexity of *ERBB2* splicing regulation in breast tumors (Fig. 2E). Many of the *ERBB2* novel isoforms alter splicing of exons encoding known protein domains. We also found multiple novel spliced isoforms of genes significantly mutated in breast cancer, including *NCOR1*, *GATA3*, *SPEN*, and *PTEN* (fig. S4B), as well as genes known to be alternatively spliced in cancer such as *CASP8*, *ENAH*, *BCL2L1*, and *STAT3* (fig. S4C). In summary, LR-seq profiling of breast tumors identifies novel spliced isoforms in genes previously associated with key cancer pathways and in known breast cancer oncogenes.

### Novel breast cancer LR-seq isoforms lead to alternative protein products

To understand the potential functional consequences of novel isoforms from our LR-seq breast cancer transcriptome at the protein level, we extracted open reading frames (ORFs; i.e., coding sequences) and predicted domains, transmembrane regions, and subcellular localization using our ORF annotation pipeline (https://brca-isoforms.jax.org/), which includes Transdecoder for ORF predictions, as well as DeepLoc, TMHMM, and hmmer for localization predictions, and in-house scripts for comparative sequence analysis and nonsense-mediated mRNA decay (NMD) predictions (Materials and Methods and fig. S1).

Overall, isoforms from all categories had very high coding potential (94 to 97%) based on our ORF prediction, except for antisense and intergenic transcripts for which 74% have a predicted ORF (fig. S5A). However, NIC and NNC spliced isoforms are more likely to be targeted for mRNA degradation by the NMD pathway, because 11% of NIC and 20% of NNC translated ORFs contain premature termination codons compared to 3% of FSM ORFs (fig. S5B). Similarly, novel ORFs absent from the protein coding database UniProt are subject to NMD at equivalent rates (11 and 22% of NIC and NNC, respectively) (fig. S5B).

**Fig. 3.**
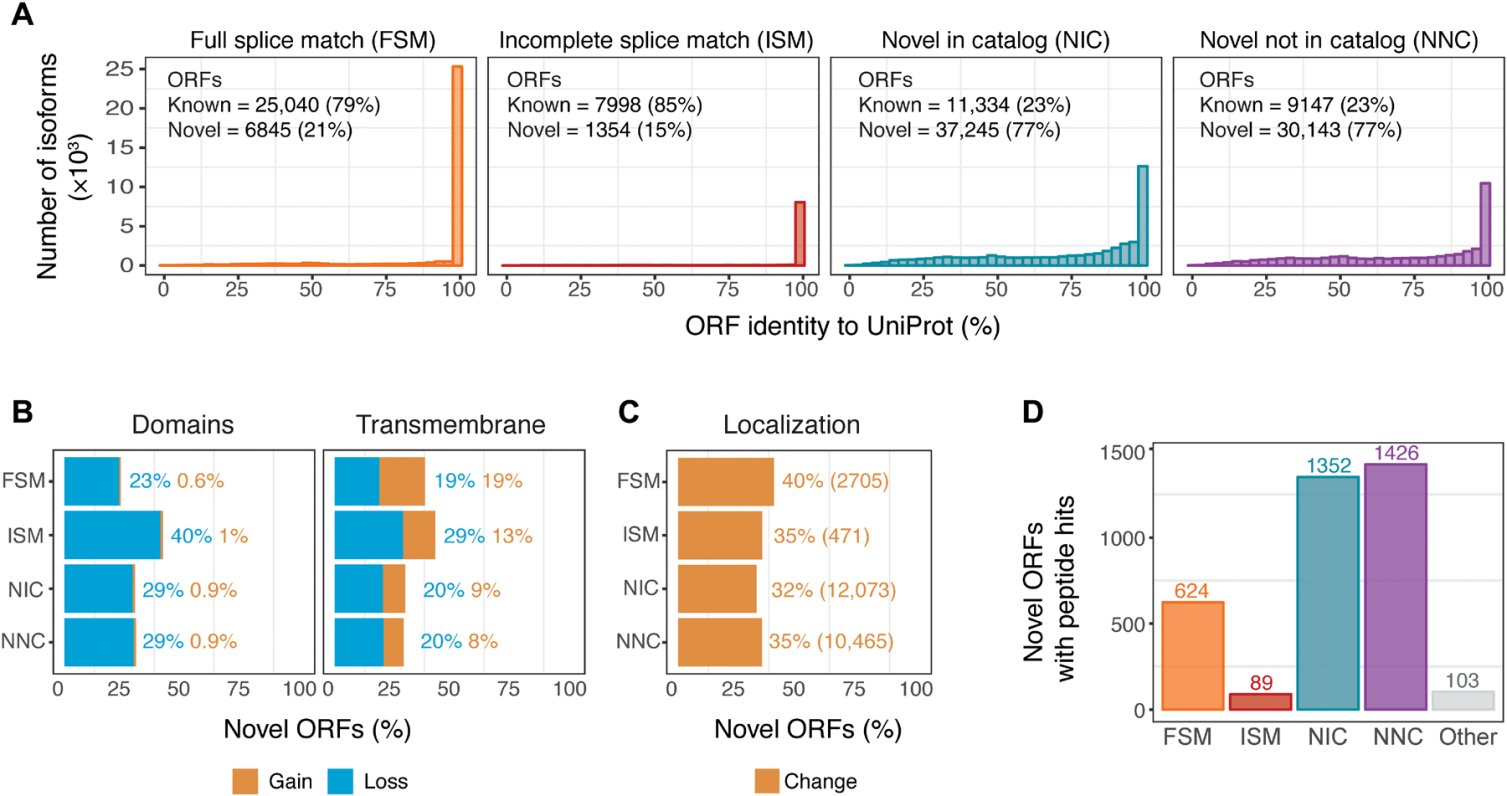
Novel LR-seq isoforms detected in breast tumors are predicted to affect protein sequence, domains, or localization. (**A**) Percent of amino acid sequence identity for LR-seq isoform–derived ORFs compared to their closest human protein isoform in UniProt, plotted by isoform structural category from Fig. 1A. Known ORFs exhibit >99% identity and unannotated ORFs <99% identity with UniProt. See also fig. S5. (**B**) Percent of novel LR-seq isoform–derived ORFs predicted to gain or lose a conserved PFAM domain or transmembrane region compared to their closest human protein isoform in UniProt. (**C**) Percent of novel LR-seq isoform–derived ORFs predicted by DeepLoc to exhibit a different subcellular localization compared to their closest human protein isoform in UniProt. The absolute number of ORFs in each structural category is indicated. See also fig. S5 (C and D). (**D**) Number of novel LR-seq isoform–derived ORFs validated by MS/MS proteomics, plotted per isoform structural category from Fig. 1A. Peptide search was conducted using 275 breast cancer samples (170 patients) from Clinical Proteomic Tumor Analysis Consortium (CPTAC).

To determine whether LR-seq isoforms encode novel protein sequences, we compared the ORF of an LR-seq isoform to its closest match in UniProt using global pairwise alignment. The majority of annotated FSM (79%) and incomplete splice match (ISM) (85%) LR-seq isoforms encode ORFs that are >99% identical to an entry in UniProt (Fig. 3A). In contrast, only 23% of NIC or NNC LR-seq isoforms are annotated in UniProt (Fig. 3A). Thus, novel LR-seq isoforms are potential sources of novel proteomic diversity in breast cancer. We then investigated whether AS in our LR-seq breast cancer transcriptome leads to novel ORFs harboring changes in annotated protein domains, transmembrane regions, or cellular localization. We found that ~20 to 30% of the novel ORFs lead to the loss of a transmembrane region or domain from the PFAM database (Fig. 3B), suggesting major changes in protein function or localization. In parallel, we used DeepLoc, a deep neural network–based tool (*27*), to predict the most likely subcellular compartment of LR-seq isoform–derived ORFs. We predicted that a third of the novel protein isoforms (25,714 ORFs) would change their subcellular localization compartment compared to their corresponding canonical UniProt entry (Fig. 3C). The localization switches are found primarily between cytoplasmic and nuclear localized protein isoforms (7580 ORFs), followed by cytoplasmic and mitochondrial changes (3777 ORFs) (fig. S5C). As an example, we next applied our isoform annotation pipeline to investigate the predicted functional effects of AS in *ESR1* (ERα), a clinical biomarker of hormone-positive breast cancers with several AS isoforms associated with cancer progression or treatment (*28*). In total, we detected 22 protein-coding isoforms in the *ESR1* locus, with 18 NIC or NNC isoforms being absent from GENCODE v.30 (fig. S5D). Among those, seven novel protein isoforms are predicted to lack the DNA binding domain. Eleven *ESR1* isoforms contained with novel regions affected by AS, including five protein isoforms with loss of the ligand-independent transactivation domain (AF1) and six protein isoforms with loss of the ligand-dependent transactivation domain (AF2) (fig. S5D). A unique *ESR1* isoform was predicted by TMHMM to contain a transmembrane domain and by DeepLoc to be localized to the cell membrane (fig. S5D). Therefore, AS in breast cancer often leads to changes in protein localization that might affect spliced isoform function.

Beyond transcript annotation, our pipeline leverages existing proteomics data for isoform validation (Fig. 3D). To determine the rate of isoform detection by tandem mass spectrometry (MS/MS) proteomics, we performed in silico peptide identification using our LR-seq–derived ORFs. We then intersected our data by spectral matching between theoretical peptides derived from LR-seq ORFs and experimentally mapped peptides from 275 publicly available breast tumors samples (170 distinct patients) profiled by MS/MS proteomics by the Clinical Proteomic Tumor Analysis Consortium (CPTAC), including 125 TCGA patients (*29*) and an additional 45 patient cohort (*30*). The proteomic analysis found isoform-specific peptides supporting 1352 NIC and 1426 NNC LR-seq–derived ORFs (Fig. 3D). In addition, we also identified 624 annotated FSM isoforms producing novel ORFs not present in UniProt.

To determine whether novel isoforms were actively translated, we performed an isoform-specific ribosome profiling analysis using Ribo-seq (ribosome profiling) data from breast cancer cell lines (*31*) and applied ORQAS (ORF quantification pipeline for AS) (*32*) to compute signal periodicity (*f1*) and uniformity (*pme*) of ribosome occupancy across known and novel ORFs. To obtain sample-specific periodicity and uniformity cutoffs that are indicative of an isoform being translated, we selected as positive controls 343 single-ORF housekeeping genes that are found to be expressed in all tissues according to the Human Protein Atlas (fig. S6A). These cutoffs were applied to the remaining of our LR-seq transcripts and found that on average 53% of known isoforms (FSM) and 36% of novel isoforms (NIC and NNC) have evidence of active translation from the Ribo-seq data in breast cancer cell lines (fig. S6B). In sum, our analytical pipeline reveals that previously unannotated spliced LR-seq isoforms detected in breast cancer encode novel protein isoforms with changes in functional domains, transmembrane regions, and/or cellular localization and that the translation of novel isoforms can be confirmed with MS/MS and ribosomal profiling data.

### Tumor subpopulations can be clustered by distinct splicing signatures

**Fig. 4.**
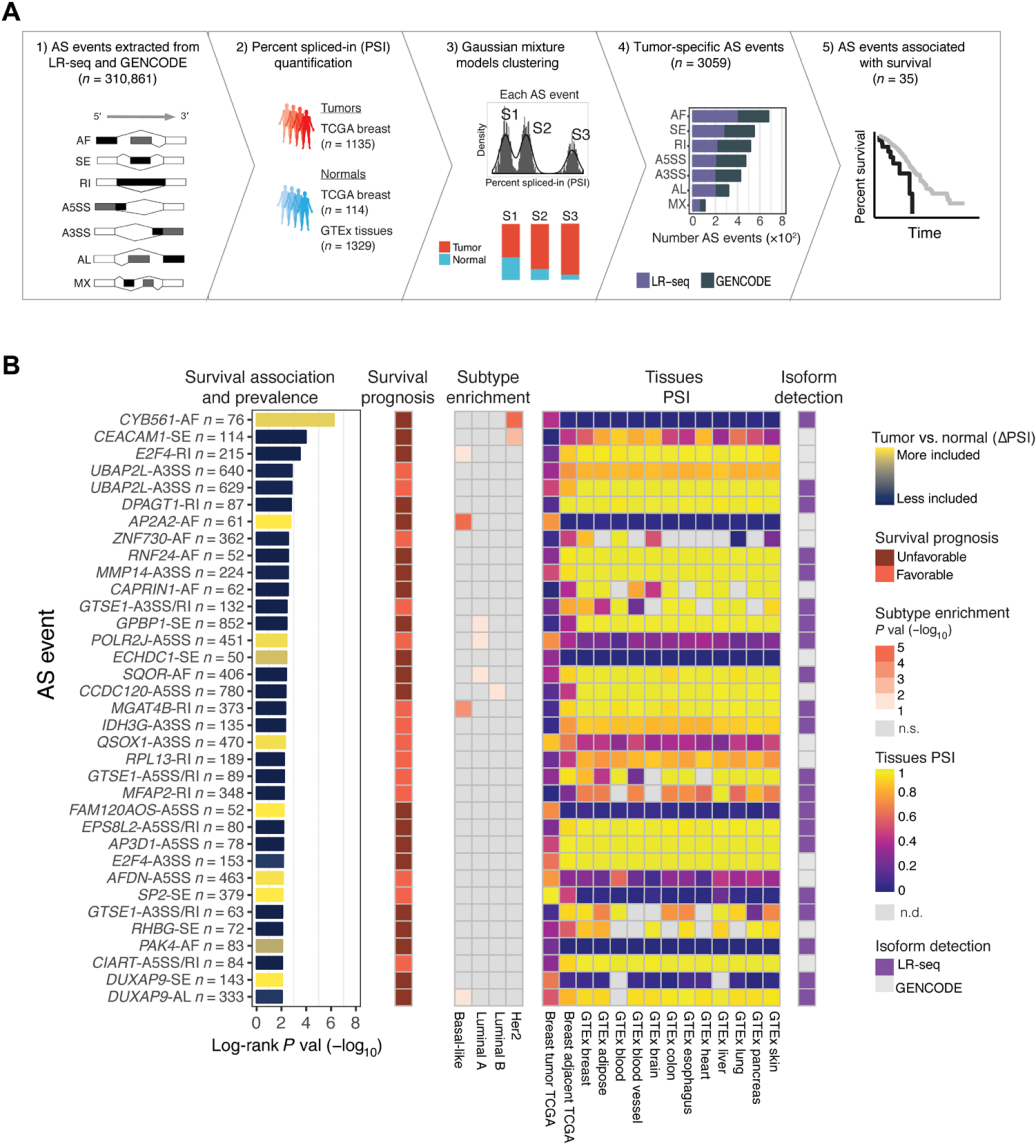
Patient clustering identifies splicing alterations associated with overall survival in breast cancer. (**A**) Identification of tumor-specific AS events in TCGA breast cancer patient subpopulations using the GMM clustering approach. Seven types of AS events (SE, MX, A5SS, A3SS, RI, AF, and AL) were extracted from both LR-seq and GENCODE isoforms (1) and quantified as PSI with SUPPA2 using RNA-seq from 2579 samples including TCGA breast tumors and normal tissues from TCGA and GTEx (2). The GMM clustering approach provided for each AS event the optimal number of distinct sample subpopulations (e.g., S1 to S3) that fit the PSI distribution, as well as the frequency of tumor and control samples in each subpopulation (3). The GMM clustering identified 3059 tumor-specific AS events in TCGA breast tumors versus normal tissues, plotted per AS event type (4). The Kaplan-Meier survival analysis compared survival rates in the identified subpopulations for tumor-specific events and detected 35 AS events associated with subpopulations with differential survival in TCGA (5). (**B**) Tumor-enriched AS events associated with overall survival in TCGA breast tumors identified by the GMM clustering approach from (A). Only AS events detected in ≥50 patients, with |ΔPSI | ≥ 20%, and with significant survival association are shown, ranked by differential survival (log-rank test, adjusted *P* < 0.01). AS events are labeled with gene name, AS event type, and number of patients and colored based on inclusion levels (ΔPSI) in tumors versus normal tissues. Information for each AS event is depicted in heatmaps, including survival prognosis, breast tumor subtype enrichment, tissues PSI values, and source of isoform detection. n.s., not significant; n.d., not detected.

To specifically identify LR-seq isoforms enriched in breast tumors versus normal tissues, we analyzed AS events in 1135 human breast tumors and 1443 normal tissue samples from TCGA and (Genotype-Tissue Expression) GTEx. We used SUPPA2 (*33*) to extract 310,861 AS events in these 2579 RNA-seq samples (Fig. 4A), using isoforms unique to our LR-seq breast cancer transcriptome (NIC and NNC) and annotated GENCODE v.30 transcripts as a reference. SUPPA2 quantifies AS events using percent spliced-in (PSI), which measures the ratio of isoforms harboring the AS event across seven types of events: skipped exon (SE), mutually exclusive exons (MX), alternative 5′ splice site (A5SS), alternative 3′ splice site (A3SS), retained intron (RI), alternative first exon (AF), and alternative last exon (AL).

Given the heterogeneity of breast cancer, which can be classified into different subtypes based on gene expression and AS levels (*8*, *34*), we introduced a novel approach for stratifying patients into groups based on distinct splicing alterations when compared to control samples. Our Gaussian mixture model (GMM) clustering approach simultaneously groups tumors and normal samples based on AS expression patterns, and then identifies, for each splicing event, several clusters (i.e., sample subpopulations) with two major features: (i) high frequency of tumor samples and (ii) significant differential splicing (ΔPSI) compared to normal tissues (Fig. 4A). Overall, our GMM clustering analysis identified 3059 tumor-specific AS events in breast cancer with |ΔPSI| ≥ 20% in subpopulations of at least 50 patients (Fig. 4A). Of those, 1638 AS events (54%) were derived from isoforms present in our LR-seq breast cancer transcriptome and not annotated in GENCODE v.30, which highlights the contribution of novel isoforms in tumor-associated splicing (Fig. 4A). Therefore, our GMM clustering approach identified recurrent AS events in breast cancer and found that they are often restricted to a subpopulation of patients in TCGA.

### Discovery of tumor-specific splicing events associated with survival

To distinguish isoforms associated with breast cancer prognosis, we directly compared the overall survival rates of each of the 3059 tumor-specific AS events identified by GMM clustering. A total of 35 AS events in 30 distinct genes correlated with survival in TCGA (Fig. 4B and file S3). The most highly associated AS events with a decrease in overall survival are an alternative first exon in *CYB561*, a skipped exon in *CEACAM1*, and loss of an intron retention event in *E2F4*. Genes containing AS events associated with overall survival are known components of cancer-related pathways, including regulation of transcription (*E2F4*, *ZNF730*, *GPBP1*, *POLR2J*, *SP2*, and *CIART*), cell cycle (*E2F4* and *GTSE1*), or cell-cell adhesion (*CEACAM1*, *MMP14*, *EPS8L2*, *AP3D1*, *AFDN*, and *PAK4*) (Fig. 4B).

Ten of the 35 overall survival-associated AS events are enriched in specific breast cancer subtypes. For example, events in *CYB561* and *CEACAM1* are enriched in *HER2*+, and *E2F4*, *AP2A2*, *MGAT4B*, and *DUXAP9* events are enriched in basal-like breast tumors (Fig. 4B). Last, 21 of our overall survival-associated AS events (~60%) were absent from GENCODE v.30, including the events in *CYB561*, *UBAP2L*, and *DUXAP9*. This indicates the importance of LR-seq in developing reference isoform transcriptomes that can elucidate clinically relevant AS events.

**Fig. 5.**
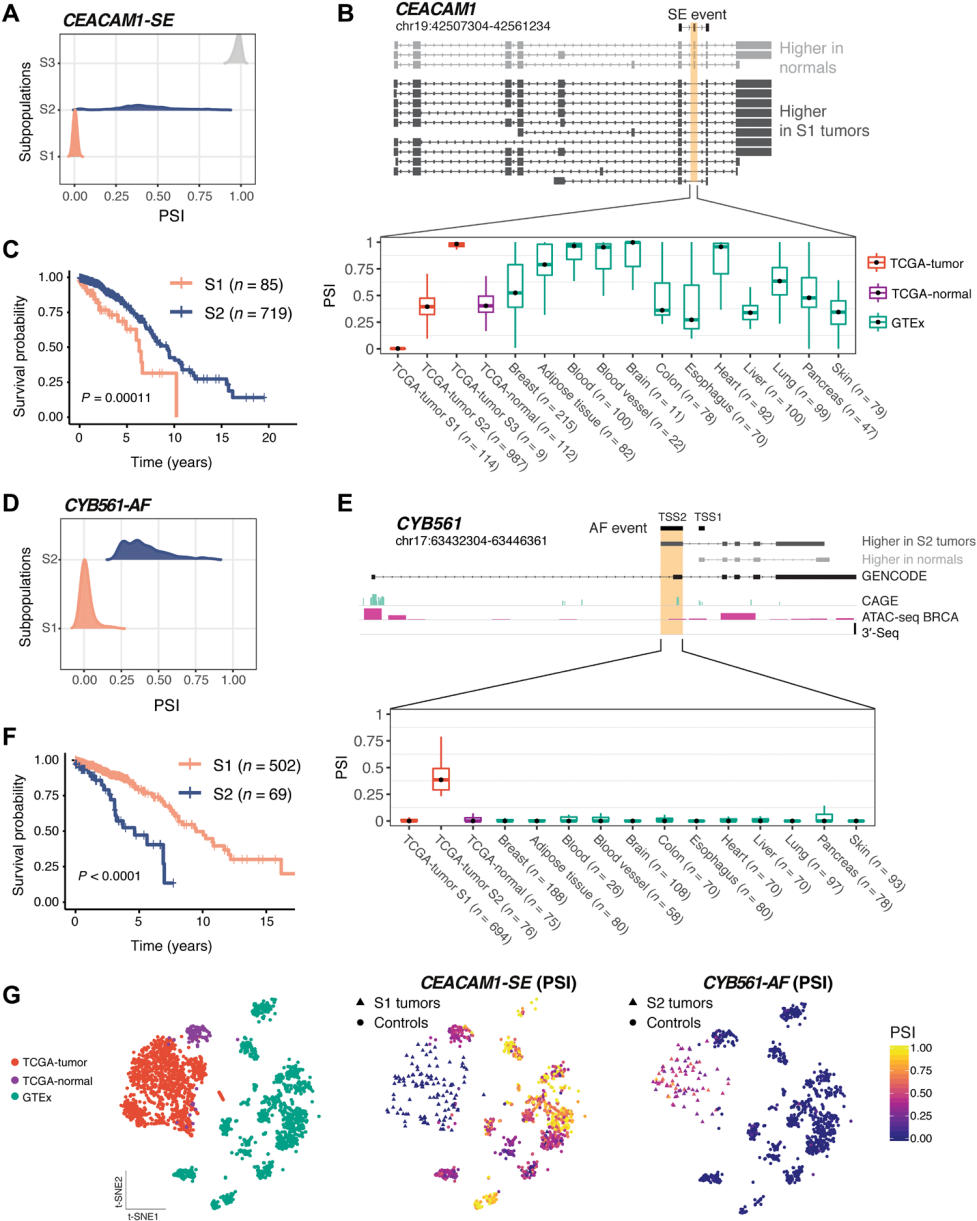
AS events in CEACAM1 and CYB561 are tumor specific and associated with unfavorable prognosis in TCGA. (**A**) TCGA tumor subpopulations (S1 to S3) detected by GMM clustering exhibit different PSI of exon 7 in *CEACAM1*. (**B**) Structure of *CEACAM1* isoforms detected by LR-seq in breast tumors or normal tissues, highlighting the location of skipped exon 7 (top). Exon 7 PSI is shown in TCGA tumor subpopulations, TCGA normal adjacent breast tissues, and GTEX normal tissues (bottom). (**C**) Overall survival in TCGA breast cancer patients in S1 subpopulation, with *CEACAM1* exon 7 skipping, and S2 subpopulation, with higher exon 7 inclusion (log-rank test). (**D**) TCGA subpopulations (S1 and S2) detected by GMM clustering exhibit different PSI values for an alternative first exon in CYB561. (**E**) Structure of *CYB561* isoforms detected by LR-seq in breast tumors or normal tissues, highlighting the location of novel (TSS1) or known alternative (TSS2) transcriptional start sites (top). CAGE, ATAC-seq, and 3′-seq genomic tracks are displayed. PSI of the isoform containing the *CYB561* TSS2 in TCGA tumor subpopulations, TCGA normal adjacent breast tissues, and GTEX normal tissues (bottom). (**F**) Overall survival in TCGA breast cancer patients in S1 subpopulation, with lower TSS2 inclusion, and S2 subpopulation, with higher TSS2 inclusion (log-rank test). (**G**) t-Distributed stochastic neighbor embedding (t-SNE) representations of the *CEACAM1* and *CYB561* AS events, showing samples per dataset (left) and colored by PSI levels for each tumor subpopulation and controls (right).

Among the AS events associated with survival differences, we identified an exon skipping event in the cell adhesion molecule *CEACAM1* in 114 TCGA breast cancer patients (Figs. 4B and 5, A to C) that was previously described in another breast cancer cohort (*35*). This AS event in *CEACAM1* affects 14 isoforms (Fig. 5B). The GMM clustering identified three tumor subpopulations (S1 to S3) with distinct exon 7 inclusion levels, revealing that the exon can be variably included or skipped in the TCGA cohort (Fig. 5A). Exon 7 of *CEACAM1* was skipped in one of the breast cancer patient subpopulations, *CEACAM1*-S1, yet was preferentially included in normal breast from TCGA, normal breast tissue from GTEx, and in several normal tissues such as lung, liver, heart, brain, blood, and adipose tissue (Fig. 5B and fig. S7A). The *CEACAM1*-S1 subpopulation with increased exon 7 skipping had worse overall survival when compared to the *CEACAM1*-S2 subpopulation (Fig. 5C), thus linking the SE event to an unfavorable disease outcome.

Our analysis also identified a breast cancer–specific AF event involving two isoforms of *CYB561*, including a novel isoform identified by LR-seq. The GMM clustering detected two tumor subpopulations with distinct transcriptional start sites, TSS1 (novel) and TSS2 (known) (Fig. 5, D and E). Patients in the *CYB561*-S2 subpopulation have a higher utilization of the isoform originating at the TSS2 start site, in comparison to the *CYB561*-S1 subpopulation and control tissues (Fig. 5E and fig. S7B). Moreover, the *CYB561*-S2 subpopulation exhibited worse overall survival when compared to the *CYB561*-S1 subpopulation (Fig. 5F), thus linking the AF event to an unfavorable disease outcome. This AF event involves a isoform not annotated in GENCODE v.30, with a start site (TSS1) supported by CAGE and ATAC-seq peaks (Figs. 4B and 5E). The detection of this AF event was only possible due to the incorporation of this novel LR-seq isoform in the disease transcriptome. *CYB561* is an electron carrier enzyme that was recently identified as a novel prognostic factor in breast cancer (*36*).

We also identified a loss of intron retention in the breast cancer oncogene *E2F4* affecting 215 (19%) of TCGA breast tumors (Fig. 4B and fig. S8). The GMM clustering identified three subpopulations with different splicing of *E2F4* isoforms in the TCGA cohort (fig. S8A). In the *E2F4*-S1 subpopulation, *E2F4* switches from an intron-containing transcript in normal tissues that is not translated to a protein coding isoform in which the intron is spliced out in breast cancer patients (fig. S8). The *E2F4* loss of intron retention is highly specific to breast cancer (Fig. 4B and fig. S8). Increased expression of the *E2F4* transcription factor is associated with cancer severity and poorer prognosis in breast cancer (*37*). In line with these findings, patients with loss of *E2F4* intron retention (*E2F4*-S1 subpopulation) have unfavorable prognosis when compared with patients where the intron is retained (fig. S8). Thus, this retained intron event might represent a regulatory mechanism by which splicing leads to up-regulation of the *E2F4* oncogene in breast cancer.

In summary, patient stratification using our novel GMM clustering analysis identified tumor-specific splicing events in breast cancer that are confined to patient subpopulations with variable prevalence. These patient subpopulations carry distinctive splice alterations compared to tumor adjacent and normal tissues (Fig. 5G and fig. S8E), and the differential splicing within these confined subpopulations could only be identified after patient stratification. We validate several of these previously undetected isoforms in breast cancer cell lines (fig. S9). In addition, our analyses implicated genes regulated by AS such as *CEACAM1*, *CYB561*, and *E2F4* as potentially playing a role in disease outcome.

## DISCUSSION

We performed LR-seq on 30 breast tumor and normal samples to define the FL isoform-level transcriptome of human breast cancers and developed an analytical pipeline to predict the functional consequences of cancer-associated splicing changes. We identified isoform-level diversity in tumors and developed a thorough LR-seq–based breast cancer isoform catalog for quantitative and qualitative assessment of potential translation and subsequent protein domain effects. The data are made available through an interactive web portal (https://brca-isoforms.jax.org) that provides tools for querying, visualizing, and downloading data. We integrated LR-seq isoforms with orthogonal datasets to demonstrate the reliability of the approach and proposed an analysis framework to detect the functional consequences of spliced isoforms in cancer. Last, we used the resultant long-read breast cancer transcriptome to uncover novel isoforms associated with patient survival in TCGA using a GMM clustering approach to identify clusters of patients with similar splicing profiles.

Our pipeline uncovers 142,514 isoforms in breast tumors, 66% of which are novel when compared with the reference transcriptome, thereby significantly increasing the repertoire of known cancer isoforms. Although short-read RNA-seq adequately supports FSM isoforms, it is unable to detect our novel NIC and NNC isoforms at similar rates, pointing to the necessity of LR-seq to accurately define isoform-level transcriptomes. Many of our novel isoforms are supported by orthogonal data, such as CAGE and ATAC-seq for transcription start sites, and 3′-seq data for 3′ untranslated regions, supporting the validity of our findings. Until now, in breast cancer, LR-seq data were available on a small number of cell lines (*13*–*15*) but not for primary tumors as described here. The proposed analysis likely captures the intertumor heterogeneity of primary tumor samples (*38*), which is absent from cell lines, and provides a more clinically relevant repertoire of spliced isoforms. This catalog of isoforms provides a more accurate and complete transcriptome enabling analyses at the isoform resolution in breast cancer and possibly other cancer types. This long-read breast cancer transcriptome will likely help the discovery of novel targets for cancer therapeutics.

Although several spliced isoforms for breast cancer genes such as *ESR1* and *ERBB2* have been previously identified (*26*, *28*), current annotations widely used for transcriptome analysis, such as RefSeq and GENCODE, do not contain the level of complexity revealed by our LR-seq analysis. A subset of the novel spliced isoforms contain distinct protein sequences, leading to novel combinations of protein domains and changes in cellular localization, and thus may play a role in promoting tumorigenesis or escaping drug response. For example, we uncovered a novel *ESR1* isoform predicted to gain a transmembrane domain and swap its localization from the nucleus to the cell membrane. Truncated ESR1 isoforms have been previously described, including several that lead to cell membrane localization (*39*–*43*). Furthermore, changes in ESR1 localization have been associated with differences in downstream signaling and in response to tamoxifen (*44*). The functional significance of the novel isoforms reported here, the predicted changes in localization, and their potential role in drug resistance remain to be experimentally defined. Although point mutations and *ESR1* amplifications have been linked with breast cancer metastasis and therapy resistance (*45*), the role of *ESR1* splicing in the tumor response to endocrine therapies remains to be determined. A systematic characterization of isoform-level variation and complexity in tumors as described here will help understand how isoforms might contribute to the heterogeneity of drug responses.

We identified AS events with prognostic value in TCGA breast cancer patients. Of 310,861 AS events detected in our LR-seq transcriptome, we found 3059 cancer-specific AS events from which 35 AS events were associated with significant changes in patient survival. This analysis reveals that while AS events are frequent in cancer transcriptomes, they are mostly restricted to subpopulations of patients. However, several AS events are recurrent and affect more than half of TCGA patients, affecting *UBAP2L*, a ubiquitin-associated protein up-regulated in breast tumors and implicated in breast cancer cell cycle control (*46*); *GPBP1*, a GC-rich promoter-binding protein previously implicated in resistance to cisplatin and poly(ADP-ribose) polymerase inhibitors in ovarian cancer (*45*); and *CCDC120*, an interaction partner of the ADP-ribosylation factor 6 that is associated with breast cancer cell invasion (*47*). The analysis also uncovered a novel regulatory mechanism by which the oncogenic E2F4 transcription factor is up-regulated in breast tumors (*37*), linking an intron retention event in *E2F4* with unfavorable prognosis in patients with breast cancer.

In conclusion, LR-seq is particularly well suited for the discovery of isoforms containing novel targets for immuno-oncology. These include the identification of cell surface isoforms against which specific monoclonal antibodies can be generated for use as therapeutics or as backbones for chimeric antigen receptor (CAR) T cells. Isoforms also generate peptides that could be used for vaccination protocols, possibly in combination with checkpoint inhibitors.

## MATERIALS AND METHODS

### Clinical samples

The study was conducted following approval by the Institutional Review Board(IRB) of The Jackson Laboratory for Genomic Medicine (IRB nos. 16-NHSR-15, 17-JGM-06, and 2018-039). Normal breast samples were acquired from the Maine Cancer Biospecimen Portal. Breast cancer tissue sections were contributed by K.P. Exempt primary tissues from patients with breast cancer were obtained from the Baylor University Medical Center (BUMC) Tissue Bank (IRB no. 005-145; otherwise discarded tissues). Consecutive postsurgical tumor samples (from patients with in situ, invasive ductal, lobular, and/or mucinous carcinoma of the breast) were collected between years 2006 and 2013.

The samples used in this research from BUMC were collected with appropriate informed consent, and the use of these samples was approved by The Jackson Laboratory (JAX) IRB (17-JGM-06). The use of deidentified samples from the Maine Cancer Biospecimen Portal and BUMC Tissue Bank was reviewed by The Jackson Laboratory (JAX) IRB (16-NHSR-15 and 2018-039) and determined to not meet the definition of human subjects research under HHS regulation 45 CFR 46.

### PDX tumor samples lines

Snap-frozen PDX tumor samples were obtained from The Jackson Laboratory (Sacramento, CA; catalog numbers provided in file S1 as tumor identifiers). Upon receipt, frozen PDX tumors were placed in cryomolds (VWR #4557), embedded in optimal cutting temperature (OCT) media (VWR #4583), and stored at −80°C before RNA extraction.

### Cell lines

Cell lines were purchased from the American Type Culture Collection (ATCC; Manassas, VA). CAMA-1, T-47D, and BT-549 lines were cultured in Dulbecco’s modified Eagle’s medium (Thermo Fisher Scientific #11965118) supplemented with 10% fetal bovine serum (Gemini Bio #100-500). Hs578t was cultured in Dulbecco’s modified Eagle’s medium (Thermo Fisher Scientific #11965118) supplemented with 10% fetal bovine serum (Gemini Bio #100-500) and bovine insulin (0.01 mg/ml; Sigma-Aldrich #I0516). Hs578Bst was cultured with Hybri-Care Medium (ATCC #46-X) and supplemented with sodium bicarbonate (1.5 g/liter; Thermo Fisher Scientific #25080094), mouse epidermal growth factor (EGF) (30 ng/ml; Thermo Fisher Scientific #PMG8043), and 10% fetal bovine serum (Gemini Bio #100-500). MCF-7 was cultured in Eagle’s minimum essential medium (ATCC #30-2003) supplemented with recombinant human insulin (0.01 mg/ml) and 10% fetal bovine serum. MDA-MB-231 and MDA-MB-468 lines were cultured in Leibovitz’s L-15 medium (ATCC #30-2008), supplemented with 10% fetal bovine serum. HCC-1500 was cultured with RPMI 1640 medium (ATCC #30-2001) supplemented with 10% fetal bovine serum (Gemini Bio #100-500). MCF-10A was cultured in MEBMTM Mammary Epithelial Cell Growth Basal Medium (Lonza) supplemented with cholera toxin (100 ng/ml; Sigma-Aldrich #C8052), 2.00 ml of Bovine Pituitary Extract (BPE) (Lonza), 0.50 ml of human epidermal growth factor (Lonza), 0.50 ml of insulin (Lonza), and 0.50 ml of hydrocortisone (Lonza). Cell lines were kept at 37°C with 5% CO_2_.

### RNA extraction

High-quality RNA was extracted from primary tumor tissues or cells. Briefly, using a Cryostat, four to five 0.3-μm tissue sections were cut from OCT-embedded tumors, mixed in 350 μl of RLT Lysis buffer containing 10% β-mercaptoethanol, and either frozen at −30°C or sent immediately for RNA extraction. For primary cells, cells were pelleted by centrifugation and then lysed with 350 μl of RLT + 10% β-mercaptoethanol. RNA was extracted using the RNeasy Mini Prep Kit following the manufacturer’s instructions (Qiagen #74106). Samples were treated with deoxyribonuclease I (Qiagen #79254) and eluted in 30 to 50 μl of ribonuclease-free water. RNA quality and quantity were assessed using a Qubit 2.0 fluorometer (Thermo Fisher Scientific), and only samples with RNA integrity number > 8.0 were selected for sequencing.

### Long-read library preparation and sequencing

Following RNA extraction, FL cDNA synthesis from poly-A–containing transcripts was performed using the Clontech SMARTer Polymerase Chain Reaction (PCR) Kit (Takara). The resulting cDNA was PCR-amplified to generate 1 to 2 μg of cDNA, and PCR products were purified using AMPure XP magnetic beads (Beckman Coulter). Size selection was performed using the Sage Science BluePippin System to remove small cDNA fragments that were preferentially sequenced by diffusion loading. Next, SMRTbell adapters were ligated to cDNA ends and purified by magnetic beads using the SMRTbell Template Prep Kit (Pacific Biosciences), followed by sequencing in a PacBio RSII or Sequel instrument. The list of clinical samples, PDXs, and cell lines sequenced in this study is provided in the file S1. File S2 provides additional information including equipment, size selection, and sequencing metrics for individual library runs.

### Short-read RNA-seq

RNA was extracted using the Qiagen RNeasy Mini Prep kit and measured using a Qubit 2.0 fluorometer (Thermo Fisher Scientific). RNA underwent quality control testing using a 2100 Bioanalyzer (RNA 6000 Pico kit, Agilent) followed by cDNA library preparation using the KAPA Stranded mRNA-Seq kit (Roche) according to the manufacturer’s instruction. Paired end sequencing was performed at 76 base pairs on each side of the DNA fragment on the Illumina NextSeq platform. In total, RNA-seq was performed for 29 tumor and control samples, with 10.7 to 142.4 million reads sequenced per sample (mean = 46.4 million).

### PCR validation of AS events associated to survival

One microgram of RNA was reverse-transcribed using the SuperScript IV First-Strand Synthesis System with both oligod(T) and random hexamer primers per manufacturer instructions (Invitrogen #18091050). Touch-down PCR was used to amplify 200 ng of cDNA with Q5 High-Fidelity DNA Polymerase and the High-GC content buffer (New England Biolabs #M0491L), and primers are listed in file S4 on a Bio-Rad T100 Thermal Cycler (Bio-Rad #1861096). PCR products were separated in 2% agarose gel stained with SYBR Safe (Invitrogen) and imaged using ChemiDoc MP Imaging System (Bio-Rad).

### Long-read data processing

Raw PacBio Iso-seq data (BAM files) were processed using the ToFU pipeline (*48*) obtained from https://github.com/PacificBiosciences/IsoSeq_SA3nUP/wiki. Briefly, the pipeline generates nonredundant FL transcripts in the following steps: (i) classify reads as FL reads and non-FL reads based on the presence of adapters and polyA signal, (ii) identify isoform clusters for each transcript using FL read(s), and (iii) polish isoform sequences by performing error correction and obtaining a final consensus transcript using both FL and non-FL reads. FL transcripts were mapped to human (hg38) and mouse (mm10) genomes using gmap (*49*), and transcripts aligned to mouse were discarded from downstream analyses (PDX samples). FL transcripts for all samples were merged using chain_samples.py from the cDNA_Cupcake tools (https://github.com/Magdoll/cDNA_Cupcake) to create a nonredundant merged transcriptome. In addition to the samples sequenced in this study, a publicly available MCF7 cell line dataset (*50*) was reprocessed and included in the merged transcriptome (see file S2).

### RNA-seq data processing

Fastq files were aligned to the hg38 genome using hisat2 v. 2.0.4 (*51*) with default options, followed by removal of duplicate reads with samtools v. 1.3.1. Bigwig files were generated using bamCoverage v.3.3.0 from deeptools2 (*52*). Xenome v.1.0 (*53*) was used to filter out mouse and ambiguous reads in PDX samples. External RNA-seq datasets were retrieved from the dbGAP database using the following accession numbers: TCGA (phs000178.v11.p8) and GTEx (phs000424.v8.p2).

### Isoform annotation and quality control

Our isoform annotation pipeline combined several tools for isoform transcript and ORF annotation as outlined in fig. S3A. Spliced isoforms were annotated with SQANTI (*54*), using GENCODE comprehensive v.30 as reference. We also used SQANTI2 (https://github.com/Magdoll/SQANTI2) to obtain a comprehensive set of quality attributes for sequenced FL reads at both transcript and junction levels, which were applied for retaining high-quality transcripts and filtering out potential artifacts as detailed below.

#### Indel correction

First, SQANTI was used to generated an indel-corrected FASTA/GTF files by realignment of FL transcripts to the human genome hg38 and to classify isoforms based on their splice patterns using GENCODE v.30 as reference. SQANTI2 was used to compute junction coverage in the Intropolis dataset and distance of TSS to CAGE peaks. In general, novel isoforms (NIC and NNC) were filtered on the basis of 3′ end reliability [no poly(A) intrapriming], noncanonical junctions or reverse-transcriptase switching (RT-switching) junctions, and splice junction read support as described below.

#### RT-switching and noncanonical junction filter

SQANTI was used to flag transcripts with noncanonical junctions or junctions possibly derived from RT-switching.

#### Read coverage filter

We used the SQANTI2 tool and the Intropolis dataset, a large compendium of RNA-seq samples containing ~21,000 human samples from the Short Read Archive (*55*), for obtaining the read support of novel splice junctions. The read coverage filter applied to novel transcripts was defined as follows: the transcript was kept if all splice junctions were covered by at least five short reads (RNA-seq from Intropolis dataset) or the transcript was detected in at least three Iso-seq samples (i.e., minimum of three FL reads).

#### Intron retention filter

Gffcompare (*51*) was used to annotate transcripts with potential intron retention (class codes *m*, *n*, *i*, and *y*).

#### Unreliable 3′ end/poly(A) intrapriming filter

In addition, to remove potential poly-A intrapriming during the reverse transcriptase reaction, the genomic 3′ end of a transcript was considered unreliable if it had all the following properties: (i) It was located more than 100 base pairs away from an annotated TTS, (ii) the adenine percentage downstream of TTS > 80%, (iii) and no overlap with the polyA site database (*56*), a catalog of high-quality and curated poly(A) sites detected by 3′-seq. On the basis of the combination of these quality attributes, we devised the following filtering strategy for each transcript category: FSM: no filtering (all included); ISM: filtering out transcripts with unreliable 3′ ends; NIC: filters based on unreliable 3′ ends, minimum read coverage, and no intron-retention; NNC: filters based on unreliable 3′ ends, minimum read coverage, no intron-retention, no junctions labeled as RT-switch, and only canonical splice sites; other (intergenic, antisense, fusion of adjacent loci): minimum read coverage, no intron-retention, no junctions labeled as RT-switch, and only canonical splice sites. Overall, 95,398 novel isoforms (41%) passed quality control and were retained for downstream analyses, in comparison to 230,425 novel isoforms originally sequenced across all samples, thus demonstrating the parsimony of the isoform selection.

### Isoform clustering

Hierarchical clustering of samples profiled by LR-seq was performed in R using the Jaccard pairwise similarity coefficient, which was defined as *Jac*(*I_A_*,*I_B_*) = *intersection*(*I_A_*,*I_B_*)/*union*(*I_A_*,*I_B_*), where *I_A_* and *I_B_* are the set of isoforms detected in given samples *A* and *B*, respectively.

### Protein-level functional characterization of long-read isoforms

We used sequence homology and domain conservation to human protein isoforms in UniProt to determine optimal coding sequences from FL LR-seq isoforms as described below. First, we assembled a comprehensive human proteome reference including both canonical (SwissProt + TrEmbl) and spliced isoforms (VarSplice) from UniProt release 2019-04, which contained 95,915 protein sequences. Possible coding sequences (ORFs) from LR-seq isoforms were predicted using Transdecoder (*57*), and local alignment using blastp (*58*) was performed against the reference proteome using options max_target_seqs = 1 and e-value = 10^−5^ to identify homologs in UniProt. Also, PFAM domains for all extracted ORFs were predicted using the hmmscan tool from hmmer v.3.1 (http://hmmer.org) using default parameters. Then, a single best ORF for each transcript was selected on the basis of significant sequence homology (blastp) and domain conservation (hmmer) to human proteins.

Next, we performed extensive annotation of coding sequences using multiple tools and custom scripts. Prediction of transmembrane helices was carried out using TMHMM, and subcellular localization was inferred using DeepLoc. Global alignment of FL coding sequences to homologs in UniProt was carried out using the Needleman-Wunsch algorithm implemented in the pairwiseAlignment function from the Biostrings package in R. Nonsense-mediated mRNA decay (NMD) analysis was performed using a custom R script using the coding sequence predicted by Transdecoder. Specifically, an FL transcript was predicted as NMD sensitive when the stop codon occurred before the terminal exon and was located more than 55 nucleotides upstream of the last splice junction. Scripts for performing global alignment and NMD prediction were implemented using mclapply (parallel package v.3.4.1).

### Peptide search

Raw MS/MS datasets from TCGA breast cancer patients (230 samples from 125 tumors) were retrieved from the CPTAC database (*29*). In addition, 45 breast cancer samples from another patient cohort (*30*) were obtained from the ProteomeXchange database, for a total of 275 proteomic samples. Peptide identification was performed using MS-GF+ version 2018.10.15 (*59*) using a sequence database that contained 165,477 ORFs derived from long-read isoforms, in addition to 95,915 human protein sequences from UniProt, and 116 contaminant sequences. The following parameters were set for database searching: Carbamidomethyl (C), iTRAQ4plex (N-term), and iTRAQ4plex (K) were specified as fixed modifications. Oxidation (M), Deamidated (NQ), Acetyl (K), and Methyl (K) were specified as variable modifications. The precursor mass tolerance for protein identification on MS was 20 ppm, and the product ion tolerance for MS/MS was 0.05 Da. Partial cleavage by trypsin was used, with up to two missed cleavages permitted. mzID profiles identified from the search engine were then pooled using the R/Bioconductor package MSnbase (*60*), and peptide-to-spectrum matches (PSMs) satisfying both spectra and peptide false discovery rate cutoffs < 1% were kept for further analysis. Last, PSMs were classified into four types, namely, unique_PacBio (peptides uniquely mapped to a single-FL isoform and not mapped to UniProt), non_unique_PacBio (peptides mapped to more than one FL isoform and not mapped to UniProt), nonunique-PacBio + UniProt (peptides mapped to both PacBio and UniProt proteins), and multigene (peptides mapped to multiple genes).

### Ribosome occupancy analysis

Ribo-seq and RNA-seq for HMEC, MCF-10A, T47D, ZR-75-1, SUM159PT, and MDA-MB-231 were obtained from Vaklavas *et al.* (*31*), accession number GSE126736. Briefly, we used ORQAS (*32*) for aligning the breast cancer Ribo-seq data to our LR-seq transcriptome (*n* = 142,514 isoforms) and to compute signal periodicity (*f1*) and uniformity (proportion of maximum entropy or *pme*) of ribosome occupancy across all known and novel ORFs. We obtained a list of 343 single-ORF housekeeping genes that are found to be expressed in all tissues from the Human Protein Atlas and computed minimum *f1* and *pme* cutoffs that classified as translated at least 80% of single-ORF housekeeping genes in each sample. ORFs with *pme* and *f1* above the cutoffs and with at Ribo-seq coverage of at least 10 reads were considered actively translated in each sample.

### Splice event extraction and quantification in TCGA and GTEx samples

AS events were extracted and quantified using SUPPA2 (*33*), based on a GTF containing long-read isoforms merged to GENCODE v.30. The input transcript expression file containing TPM (transcripts per million) abundances of all isoforms for SUPPA2 was computed using StringTie v.1.3.0 (*51*) using the merged GTF as a reference for transcript quantification. Alternative first and last exons were defined using 250 base pairs overlap threshold (*−t* 250) for PSI calculation. For other types of events (SE, MX, RI, A3, and A5), the overlap threshold was set to 10 base pairs.

### Gaussian mixture clustering and survival analysis

The GMM clustering was implemented in R using the mclust package v. 5.4.1. The clustering approach consisted in fitting a mixture of Gaussian distributions to PSI values from AS events simultaneously in cancer and control samples, using the PSI matrix obtained from SUPPA2. Model fitting with mclust was performed using one to three Gaussian distributions (i.e., minimum of one and maximum of three PSI subpopulations), and the optimal fitting was determined using the Bayesian information criterion. For each AS event, samples were assigned to clusters (subpopulations) with highest probability, and the frequency of tumor and controls was computed within subpopulations. Subpopulations with high tumor purity (>90% breast tumor samples) were further analyzed for differential splicing and survival as described below. The Wilcox rank-sum test in R was used to determine differential splicing between a tumor-specific subpopulation and control tissues from TCGA and GTEx. Next, survival analysis was done using the pairwise_survdiff function from the survminer package v. 0.4.6, which performs pairwise comparisons between GMM-inferred subpopulations with corrections for multiple testing. Only subpopulations with at least 30 patients were included in the Kaplan-Meier analysis. Significant survival events were selected on the basis of the global *P* value and pairwise comparisons (adjusted *P* < 0.01).

### t-SNE visualization of AS events

t-Distributed stochastic neighbor embedding (t-SNE) was performed using the Rtsne package v. 0.13. The t-SNE representation was generated on the basis of the PSI matrix of exon skipping events. The PSI matrix was filtered to remove events with more than 80% of missing values. Samples with more than 80% of missing values were also removed. Missing values occurred for events in which neither the inclusion nor skipping forms are detected. Any remaining missing values were mean imputed. t-SNE with learning rate of 200 and perplexity of 50 was applied for all visualizations.

## Notes

View/request a protocol for this paper from *Bio-protocol*.
