# Supplementary material for: A comprehensive long-read isoform analysis platform and sequencing resource for breast cancer
Source: Sci Adv. 2022 Jan 19;8(3):eabg6711. doi: 10.1126/sciadv.abg6711 (PMC8769553; doi:10.1126/sciadv.abg6711)
Supplement: Supplementary file 1 — Figs. S1 to S9 Table S1 Legends for files S1 to S4 [file sciadv.abg6711_sm.pdf]

Science Advances
AAAS

Supplementary Materials for

A comprehensive long-read isoform analysis platform and sequencing resource for breast cancer

Diogo F. T. Veiga*, Alex Nesta*, Yuqi Zhao, Anne Deslattes Mays, Richie Huynh, Robert Rossi, Te-Chia Wu, Karolina Palucka, Olga Anczukow*, Christine R. Beck*, Jacques Banchereau*

*Corresponding author. Email: olga.anczukow@jax.org (O.A.); christine.beck@jax.org (C.R.B.); jacques.banchereau@gmail.com (J.B.)

Published 19 January 2022, *Sci. Adv.* **8**, eabg6711 (2022)
DOI: 10.1126/sciadv.abg6711

The PDF file includes:

Figs. S1 to S9
Table S1
Legends for files S1 to S4

Other Supplementary Material for this manuscript includes the following:

Files S1 to S4

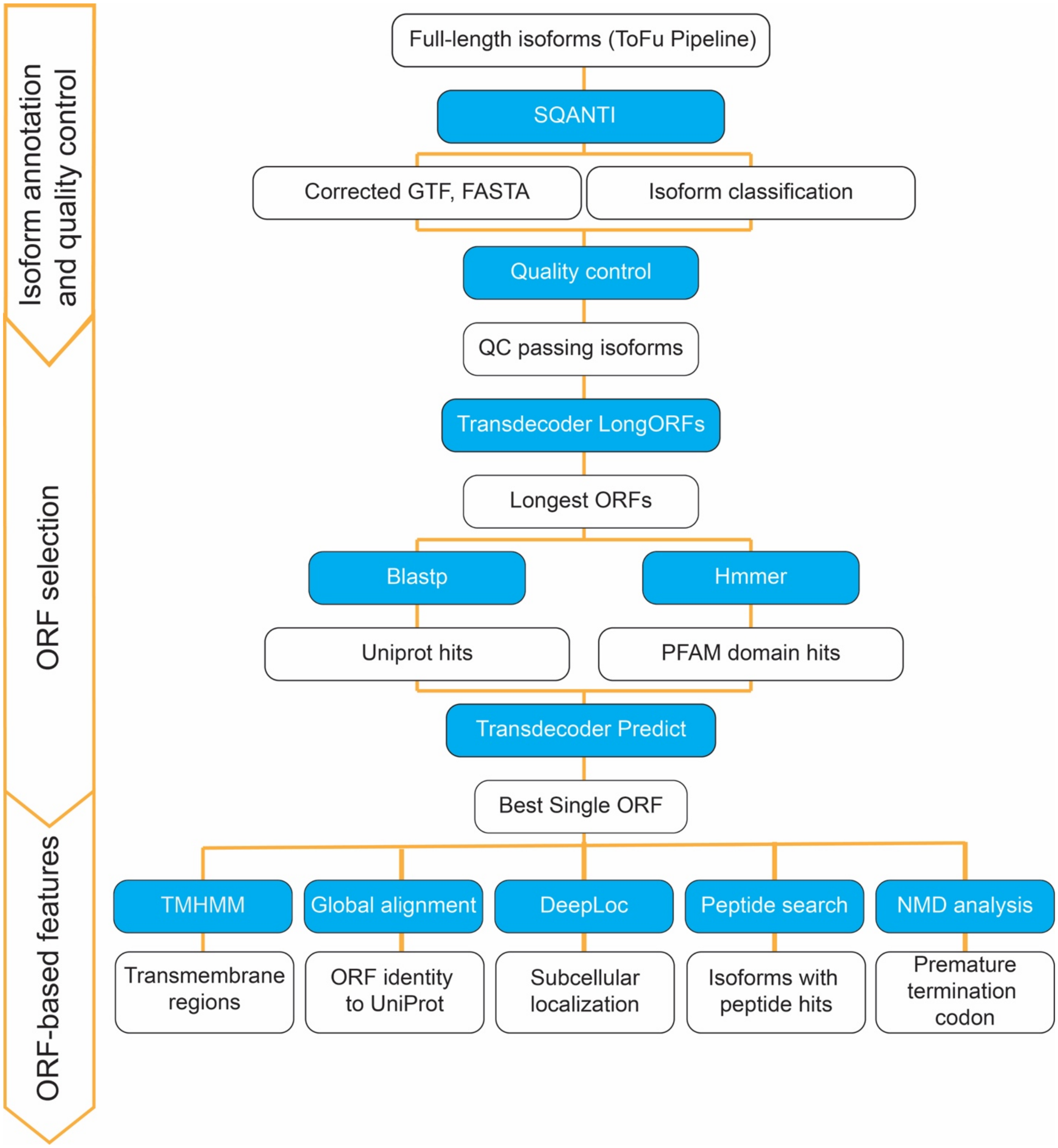

Fig. S1. Analysis pipeline for LR-seq isoform annotation, quality control and prediction of protein features (related to Fig. 1).

The pipeline is divided in three phases*:* 1) isoform annotation and quality control, 2) ORF selection, and 3) prediction of ORF-based protein features. In the first phase, SQANTI and SQANTI2 are used to obtain quality metrics for LR-seq isoforms at both transcript and junction

levels, which are applied for removing low quality long-reads containing inadequate splice junction support, and those that contained signatures of poly(A) intra-priming or non-canonical junctions derived from reverse transcriptase template switching. Second, the optimal ORF selection is performed using a combination of Transdecoder, blastp and hmmer tools. The third phase consists of downstream analysis such as global alignment of ORFs to UniProt, inference of non-mediated decay (NMD), peptide search in proteomics datasets, and prediction of protein features using TMHMM and DeepLoc. Blue boxes refer to tools and scripts used for data processing, while white boxes refer to inputs and outputs. See Methods for additional details.

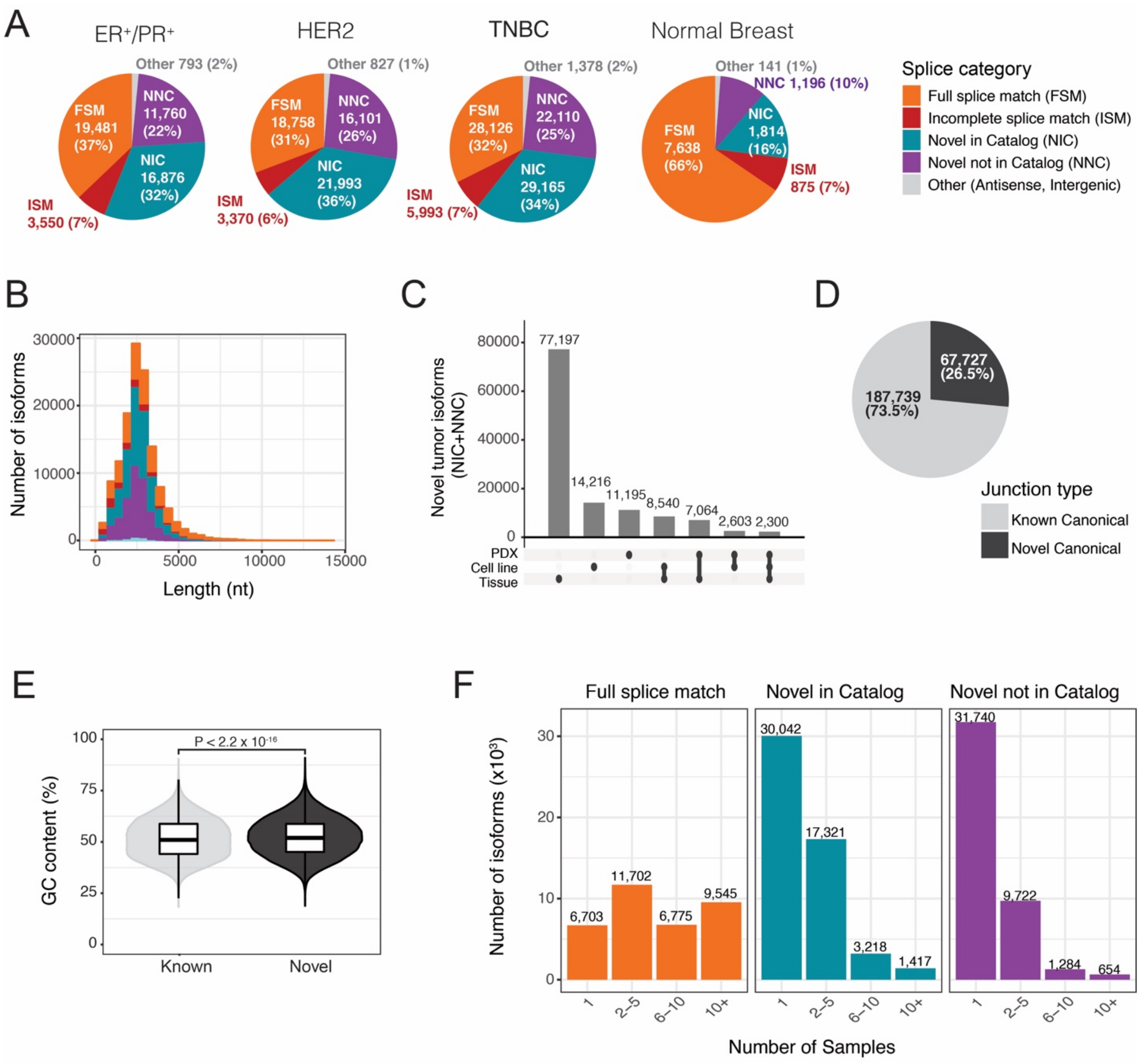

Fig. S2. Characteristics of isoforms and splice junctions detected by LR-seq (related to Fig. 1).

(A) Classification of isoforms obtained by LR-seq in samples from each breast cancer subtype (HER2+, ER+/PR+, TNBC) or normal. Isoforms are classified into structural categories as described in Fig. 1A.

(B) Length distribution (nt) of LR-seq isoforms detected, colored by structural category as described in A.

(C) Origins of novel tumor isoforms in breast cancer (NIC + NNC) according to tissue source (primary tissue, PDX and cell lines).

(D) Frequency and absolute number of novel and known unique splice junctions detected in the LR-seq breast cancer transcriptome.

(E) GC content of known or novel canonical splice junctions detected in the LR-seq breast cancer transcriptome in a region -50bp to +50bp from the splice site. The 2% increase in the mean GC content surrounding novel junctions is significant by a Wilcox rank sum test ($P < 2.2 \times 10^{-16}$).

(F) Frequency of detection of LR-seq isoforms across libraries, colored by structural category as described in A.

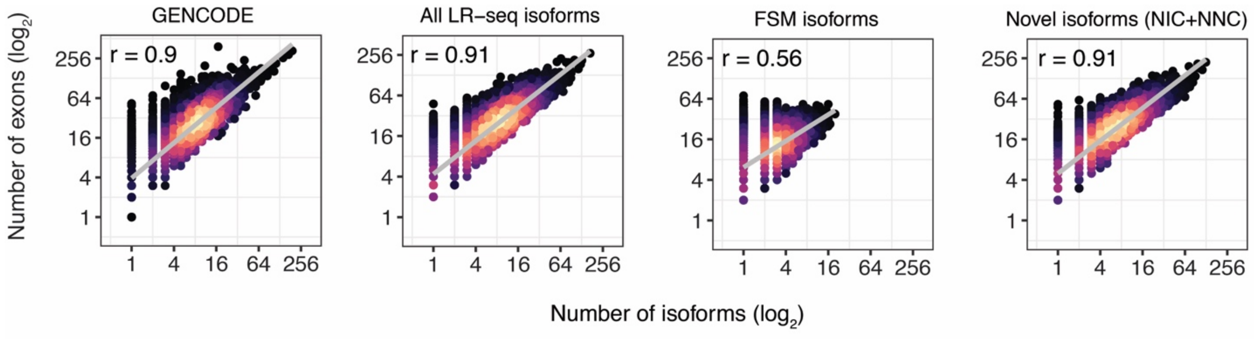

Fig. S3. Correlation between number of exons and number of LR-seq isoforms (related to Fig. 1). From left to right: isoforms in GENCODE v.30, all LR-seq isoforms detected in this study, FSM isoforms only, and novel isoforms only (NIC and NNC). Spearman correlations are indicated. See Fig.1A for FSM, NIC and NNC definitions.

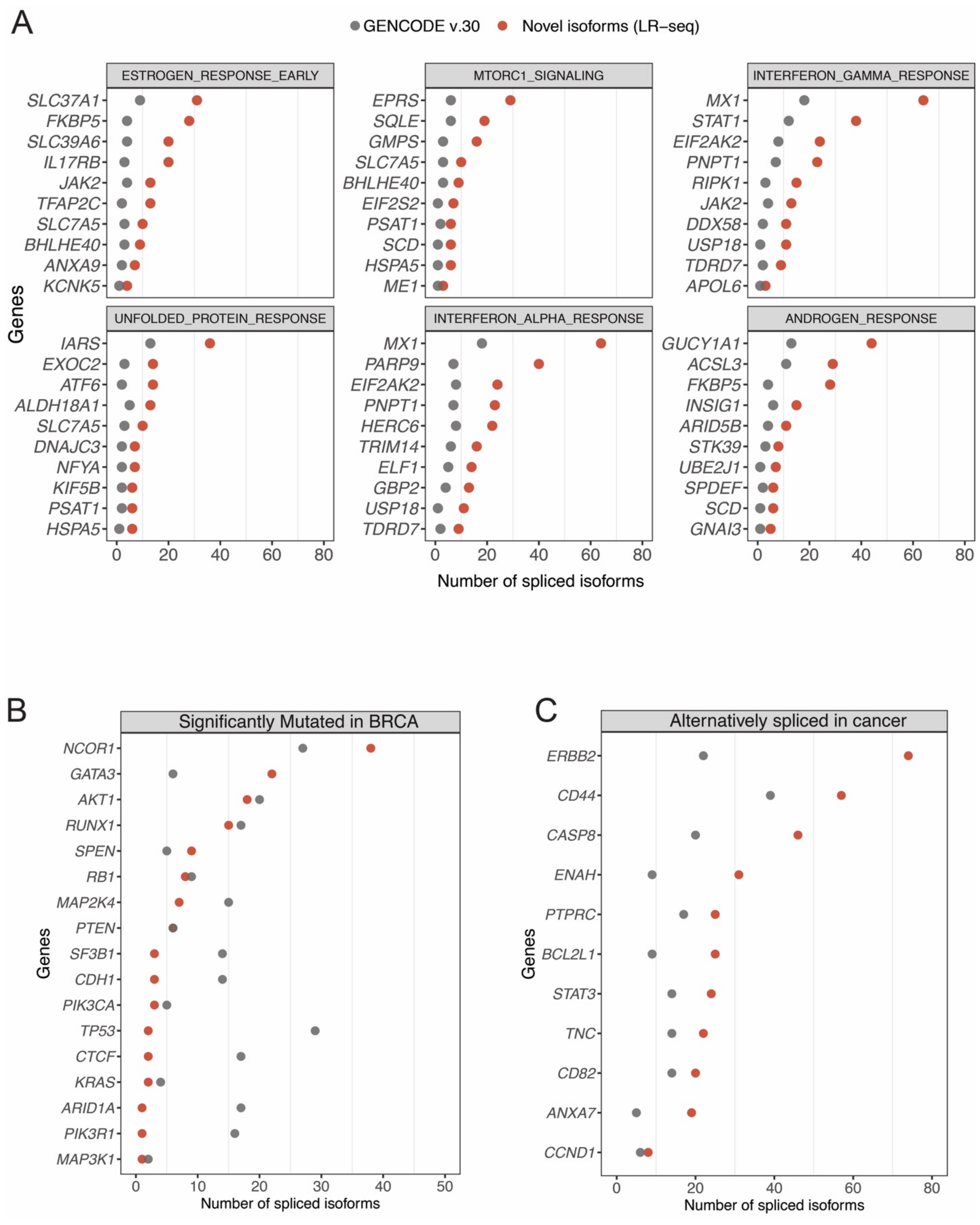

Fig. S4. Isoform increase in cancer-associated genes and pathways (related to Fig. 2).

(A) The top ten genes with the highest number of novel LR-seq isoforms are shown for each pathway from Fig. 2B compared to known isoforms from GENCODE v.30.

(B) Number of novel isoforms detected by LR-seq *vs.* GENCODE v.30 in significantly mutated breast cancer genes (http://www.tumorportal.org).

(C) Number of novel isoforms detected by LR-seq *vs.* GENCODE v.30 in genes that have been previously reported to undergo AS in cancer *(19, 20)*.

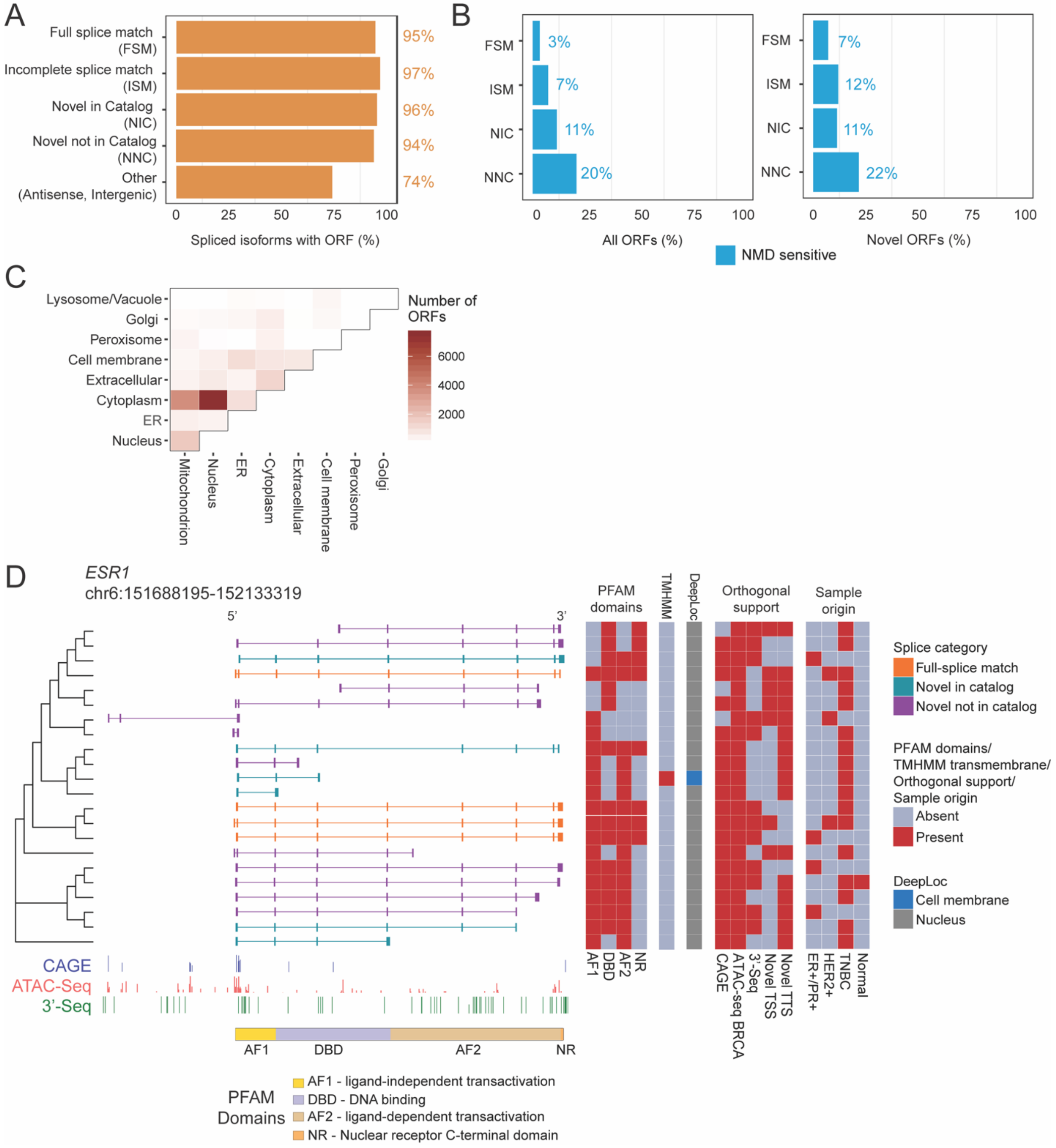

Fig. S5. Coding potential and NMD prediction of ORFs extracted from LR-seq isoforms detected in breast tumors (related to Fig. 3).

(A) Percent of LR-seq isoforms predicted by Transdecoder to contain an ORF, plotted per isoform structural category from Fig. 1A.

(B) Percent of LR-seq isoforms classified as NMD sensitive (containing a premature termination codon before the last exon) per isoform structural category for all ORFs or from novel-only ORFs. Novel ORFs have < 99% sequence similarity to their closest human protein variant in UniProt.

(C) DeepLoc-predicted changes in cellular localization for ORFs derived from LR-seq isoforms, compared to their closest human protein isoform in UniProt. The scale indicates the number of LR-seq isoforms changing their localization between each cellular localization pair.

(D) LR-seq *ESR1* isoforms detected in breast tumors and predicted changes in protein domains and localization. LR-seq isoforms are grouped based on ORF similarity. For each isoform, the presence of PFAM domains, shown schematically at the bottom (AF1 – ligand-independent transactivation domain; DBD – DNA binding domain; AF2 – ligand-dependent transactivation domain; NR – Nuclear receptor C-terminal domain), as well as transmembrane regions (TM) identified by TMHMM, and predicted subcellular localization (DeepLoc) are indicated in adjacent heatmaps. Supporting orthogonal data such as the presence or absence of CAGE or ATAC-seq peaks (ATAC-seq BRCA) supporting novel transcription start sites (TSS), or 3´-seq peaks supporting novel transcription termination sites (TTS) is indicated. The sample origin where the isoform is detected, including breast cancer subtype (ER^+/PR^+, $HER2^+$, TNBC) is indicated. Genomic tracks for CAGE, ATAC-seq and 3´-seq along with the localization of *ESR1* protein domains are displayed at the bottom.

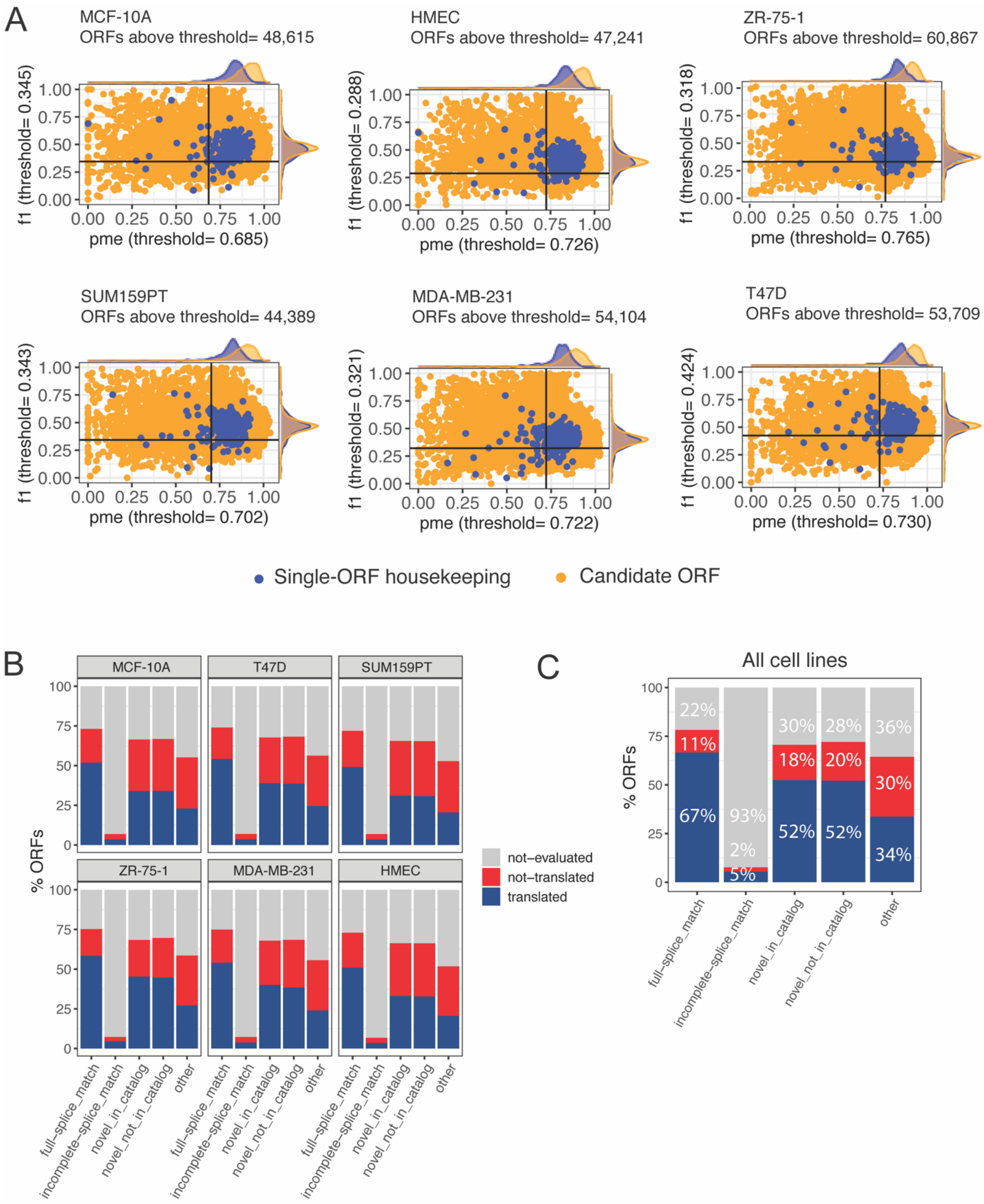

Figure S6. Ribosome occupancy analysis of LR-seq isoforms using Ribo-seq datasets from breast cancer cell lines (related to Fig. 3).

(A) Periodicity (f1) and uniformity (pme) of LR-seq ORFs in breast cancer and non-transformed mammary cell lines computed by ORQAS. Single-ORFs housekeeping genes (blue) are positive controls used to determine f1 and pme cutoffs indicative of active translation in each cell line. The number of actively translated ORFs above the cutoffs (upper right quadrant) is indicated in each cell line.

(B) Translation status of LR-seq ORFs in cell lines using the sample-specific cutoffs as shown in A.

(C) Translation status of LR-seq ORFs across all analyzed cell lines. An ORF was considered translated if detected in at least one cell line.

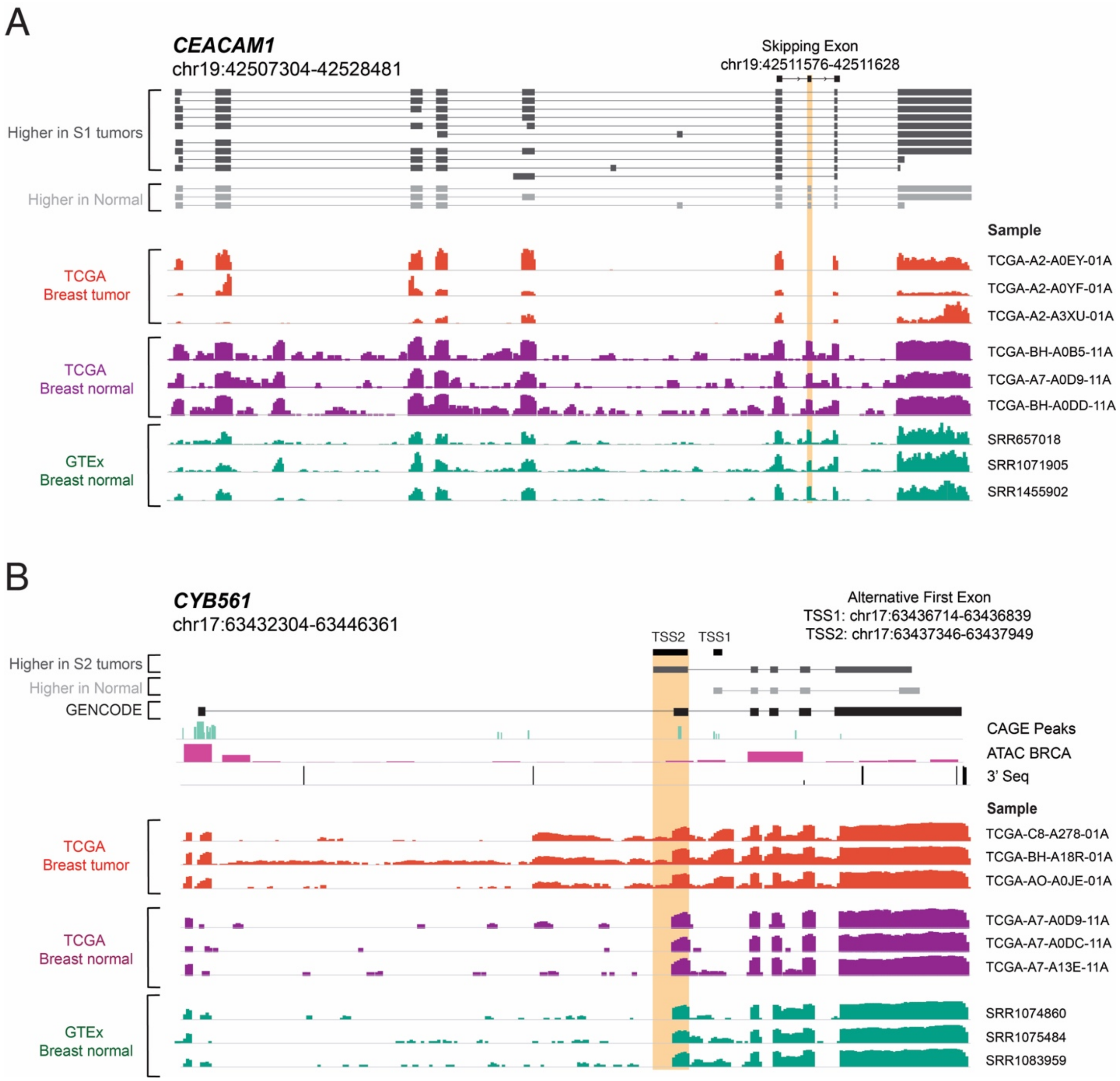

Figure S7. RNA-seq coverage plots of AS events in *CYB561* and *CEACAM1* in tumors and normal tissues (related to Fig. 5).

(A) RNA-seq coverage tracks from the *CEACAM1* isoform associated with skipped exon 7 (gene coordinates are indicated) are shown for selected TCGA (n=3), as well as normal breast tissue from TCGA (n=3) and from GTEx (n=3). Structure of *CEACAM1* isoforms detected by LR-seq in breast tumors (dark grey) or normal tissues (light grey), highlighting the location and genomic

coordinates of the skipped exon 7. TCGA and GTEX sample identification numbers are listed next to each track.

(B) RNA-seq coverage tracks from the *CYB561* isoform associated with an alternative first exon (gene coordinates are indicated) are shown for selected TCGA breast tumors (n=3), as well as normal breast tissue from TCGA (n=3) and from GTEx (n=3). Structure of *CYB561* isoforms detected by LR-seq in breast tumors (dark grey) or normal tissues (light grey), highlighting the location and genomic coordinates of known (TSS1) and novel (TSS2) transcriptional start sites. CAGE, ATAC-seq and 3'-seq genomic tracks are displayed underneath isoform structures. TCGA and GTEX sample identification numbers are listed next to each track.

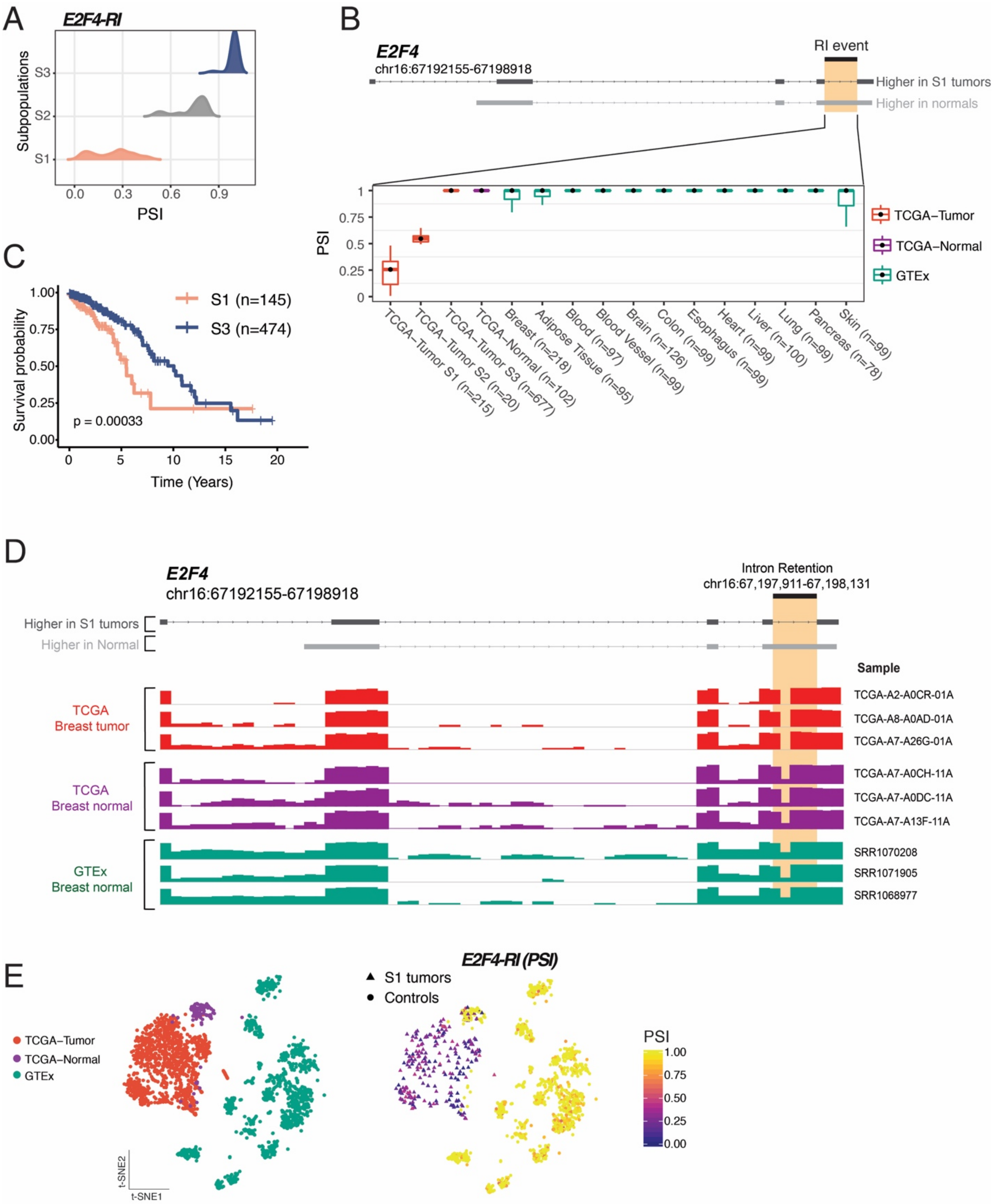

Figure S8. Loss of intron retention in *E2F4* in a tumor subpopulation correlates with unfavorable prognosis (related to Figs. 4 and 5).

(A) TCGA subpopulations (S1-S3) detected by GMM clustering express different inclusion levels (percent spliced in or PSI) of an intronic region in *E2F4*.

(B) Structure of *E2F4* isoforms detected by LR-seq in breast tumors (dark grey) or normal tissues (light grey), highlighting the location of the retained intron (B, top panel). *E2F4* intron retention (as PSI) is shown in TCGA tumor subpopulations S1, S2, and S3, as well as in normal adjacent breast tissue from TCGA and a panel of GTEX normal tissues (B, bottom panel).

(C) Kaplan-Meier analysis of overall survival in TCGA breast cancer patients in the S1 subpopulation, which exhibits lower intron retention (n=145), and the S3 subpopulation, which exhibits higher intron retention (n=474) (log-rank test, $P<0.0003$) (C).

(D) RNA-seq coverage tracks from the *E2F4* isoform associated with a retained intron event (gene coordinates are indicated) are shown for selected TCGA breast tumors (n=3), as well as normal breast tissue from TCGA (n=3) and from GTEx (n=3). Structure of *E2F4* isoforms detected by LR-seq in breast tumors (dark grey) or normal tissues (light grey), highlighting the location and genomic coordinates of retained intron. TCGA and GTEX sample identification numbers are listed next to each track.

(**E**) t-SNE representations of the AS event in *E2F4.* Left, Samples are colored according to the dataset (Breast TCGA-tumor, Breast TCGA-Normal and GTEx). Right, Samples are colored according to the PSI levels of intron retention in *E2F4.* Tumor subpopulation with differential splicing is depicted in triangles, and controls are shown in circles.

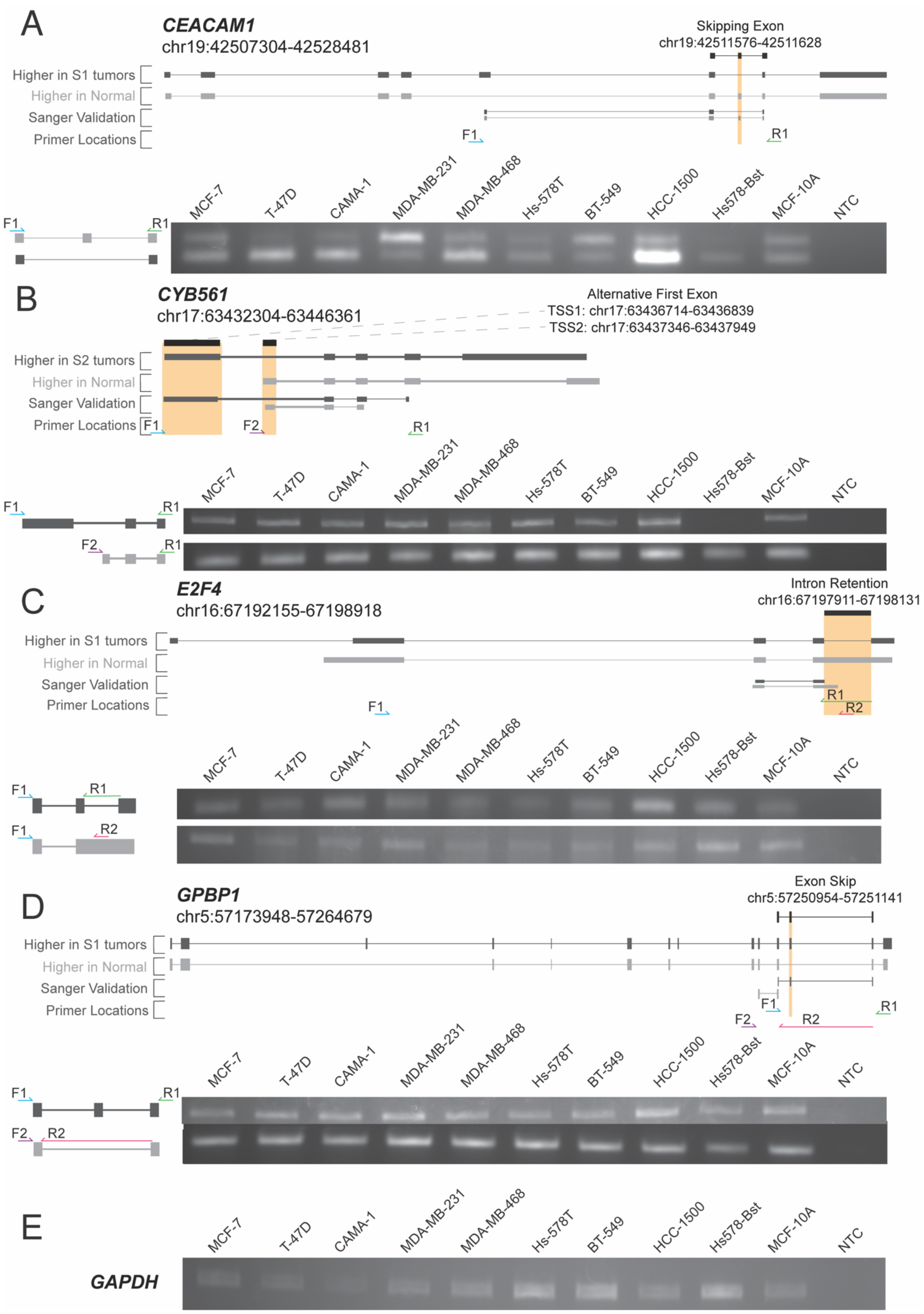

A
CEACAM1
chr19:42507304-42528481
Skipping Exon
chr19:42511576-42511628
Higher in S1 tumors
Higher in Normal
Sanger Validation
Primer Locations
F1
R1
MCF-7
T-47D
CAMA-1
MDA-MB-231
MDA-MB-468
Hs-578T
BT-549
HCC-1500
Hs578-Bst
MCF-10A
NTC
B
CYB561
chr17:63432304-63446361
Alternative First Exon
TSS1: chr17:63436714-63436839
TSS2: chr17:63437346-63437949
Higher in S2 tumors
Higher in Normal
Sanger Validation
Primer Locations
F1
F2
R1
C
E2F4
chr16:67192155-67198918
Intron Retention
chr16:67197911-67198131
Higher in S1 tumors
Higher in Normal
Sanger Validation
Primer Locations
F1
R1
R2
D
GPBP1
chr5:57173948-57264679
Exon Skip
chr5:57250954-57251141
Higher in S1 tumors
Higher in Normal
Sanger Validation
Primer Locations
F1
F2
R1
R2
E
GAPDH

Figure S9. Detection in breast cancer cell lines of the tumor-specific AS events associated with unfavorable prognosis identified in TCGA breast tumors (related to Figs. 4 and 5).

(A-D) RT-PCR detection of AS events in *CEACAM1* (A), *CYB561* (B), *E2F4* (C), or *GPBP1* (D), in breast cancer (MCF-7, T-47D, CAMA-1, MDA-MB-231, MDA-MB-468, Hs-578T, BT-549, HCC-1500) or non-transformed mammary epithelial (Hs578-Bst, MCF-10A) cell lines using isoform specific primers that amplify both the included and skipped isoforms detected by SUPPA in TCGA breast tumors. For each event, the gene coordinates, the structure of isoforms detected by LR-seq in breast tumors (dark grey) or normal tissues (light grey), highlighting the type, location and coordinates of the AS event are indicated in the top panel; an orange box highlights the differentially spliced sequence. The Sanger validation tracks show the alignment of the amplified product for a pooled set of breast cancer cell lines. The primer locations track depicts that PCR primers positions. A representative gel is shown along with the isoform structures and primer locations. NTC- no template control.

(E) RT-PCR detection of housekeeping GAPDH transcript as a loading control.

Table S1. Summary of LR-seq isoforms according to sample origin.

Classification	Origin	Samples	FSM	ISM	NIC	NNC	Other	Total isoforms
**Breast tumor**	Tissue	13	28,023 (25%)	6,669 (6%)	42,176 (37%)	35,021 (31%)	1,963 (1%)	113,852 (100%)
	Cell Line	4	16,204 (48%)	3,096 (9%)	8,825 (26%)	5,391 (16%)	424 (1%)	33,940 (100%)
	PDX	9	14,485 (53%)	1,419 (5%)	6,911 (25%)	4,284 (16%)	290 (1%)	27,389 (100%)
**Normal breast**	Tissue	2	3,923 (64%)	296 (5%)	1,112 (18%)	712 (12%)	91 (1%)	6,134 (100%)
	Cell Line	2	4,921 (71%)	636 (9%)	783 (11%)	573 (8%)	62 (1%)	6,975 (100%)

Supplementary Files

Supplementary file 1. Histology and demographics of breast cancer clinical samples, PDXs and cell lines profiled by LR-seq in this study. Provided as file "Supp. file 1- Samples.xlsx".

Supplementary file 2. Sequencing metrics, equipment, and size selection parameters for individual PacBio library runs. Provided as file "Supp. file 2- PacBio libraries.xlsx".

Supplementary file 3. Alternative splice events and associated isoforms with survival correlation in TCGA breast cancer patients. Provided as file "Supp. file 3- Survival associated AS events.xlsx".

Supplementary file 4. Primers for PCR validation of AS events in *CYB561*, *CEACAM1*, *E2F4*, and *GPBP1*. Provided as file "Supp. file 4- Primers.xlsx".
